# Supplementary material for: Prevalence and correlates of prescription drug diversion and misuse among people living with HIV in the eThekwini district, KwaZulu-Natal, South Africa
Source: PLoS One. 2020 Dec 16;15(12):e0243718. doi: 10.1371/journal.pone.0243718 (PMC7744047; doi:10.1371/journal.pone.0243718)
Supplement: S1 Table — (DOCX) [file pone.0243718.s001.docx]

**Supplementary information**

**S1 Table . Characteristics participants for the study.** This is the S1 Table legend.

| **Participant characteristics** | **Total (N=392)** |
| --- | --- |
|  | **N (%) or mean (SD)** |
| **Sex** |  |
| Male | 129 (33) |
| Female | 263 (67) |
| **Average age, years** | 36.8 (± 10.0) |
| **Marital status** |  |
| Married | 40 (10) |
| Single | 335(86) |
| Divorced | 3 (0.8) |
| Widowed | 10 (2.6) |
| Prefer not to answer | 2 (0.5) |
| **Race** |  |
| African | 388 (99) |
| Asian/Indian | 1 (0.3) |
| Coloured | 1 (0.3) |
| White | 2 (0.5) |
| **Education** |  |
| Completed high school and tertiary studies | 40 (10) |
| Completed only high school | 114 (29) |
| Not completed high school | 238 (61) |
| **Employment** |  |
| Employed | 209 (53) |
| Unemployed | 183 (47) |
| **Homeless in the past 30 days** |  |
| Yes | 17 (4) |
| No | 374 (96) |
| **Received income past month** |  |
| Yes | 300 (77) |
| No | 90 (23) |
| **Currently prescribed any prescription drugs** |  |
| Yes | 383 (98) |
| No | 9(2) |
| **Currently prescribed ARVs** |  |
| Yes | 320 (82) |
| No | 72 (18) |
| **Diagnosed with other diseases** |  |
| Yes | 118 (30) |
| No | 274 (70) |
| **Health** |  |
| Excellent | 79 (20) |
| Very good | 63 (16) |
| Good | 163 (42) |
| Fair | 36 (9) |
| Poor | 50 (13) |
| **Type of neighbourhood** |  |
| Orderly | 224 (57) |
| Disorderly, violent, crime or drug ridden | 168 (43) |
| **Medication perceived harmful** |  |
| Yes | 320 (82) |
| No | 66 (17) |
| Not sure | 3 (1) |
| **Geographical setting** |  |
| Rural | 25 (6) |
| Urban | 226 (58) |
| Semi-urban | 141 (36) |
