# Supplementary material for: Prevalence and correlates of prescription drug diversion and misuse among people living with HIV in the eThekwini district, KwaZulu-Natal, South Africa
Source: PLoS One. 2020 Dec 16;15(12):e0243718. doi: 10.1371/journal.pone.0243718 (PMC7744047; doi:10.1371/journal.pone.0243718)
Supplement: S2 Table — (DOCX) [file pone.0243718.s002.docx]

**S2 Table. Correlates of prescription drug diversion adjusted for geographical setting among PLWH in eThekwini district, participants who ever diverted prescription drugs, N = 51.** This is the S2 Table legend.

| **Variables** | **MODEL A** | | | **MODEL B** | |
| --- | --- | --- | --- | --- | --- |
|  | **Odds ratio (95% CI)** | ***p* value** | **Adjusted odds ratios (95% CI)** | | ***p* value** |
| Age in years |  |  |  | |  |
| 18 – 24 years | Reference | Reference | Reference | | Reference |
| 25 – 29 years | 0.46 (0.12 – 1.77) | 0.256 | 0.41 (0.10 – 1.73) | | 0.228 |
| 30 + years | 0.97 (0.32 – 2.94) | 0.952 | 1.03 (0.31 – 3.47) | | 0.957 |
| Gender |  |  |  | | - |
| Female | Reference | Reference | Reference | | Reference |
| Male | 2.01 (1.10 – 3.65) | **0.022** | 1.33 (0.63 – 2.81) | | 0.453 |
| Diagnosed with other conditions |  |  |  | |  |
| Has other conditions | Reference | Reference | - | | - |
| No other conditions | 1.05 (0.55 – 2.01) | 0.878 | - | | - |
| Education |  |  |  | |  |
| Completed high school | Reference | Reference | Reference | | Reference |
| Did not complete high school | 1.34 (0.72 – 2.49) | 0.359 | 1.05 (0.54 – 2.02) | | 0.891 |
| Employment status |  |  |  | |  |
| Employed | Reference | Reference | - | | - |
| Unemployed | 1.23 (0.68 – 2.22) | 0.493 | - | | - |
| Received income past month |  |  |  | |  |
| Received income | Reference | Reference | Reference | | Reference |
| No income | 2.02 (1.08 – 3.80) | **0.029** | 1.80 (0.88 – 3.66) | | 0.106 |
| Homeless |  |  |  | |  |
| Never homeless past 30 days | Reference | Reference | - | | - |
| Ever been homeless past 30 days | 1.28 (0.35 – 4.60) | 0.710 | - | | - |
| Alcohol use |  |  |  | |  |
| Never used alcohol | Reference | Reference | - | | - |
| Ever used alcohol | 1.35 (0.74 – 2.45) | 0.328 | - | | - |
| Tobacco use |  |  |  | |  |
| Never used tobacco | Reference | Reference | Reference | | Reference |
| Ever used tobacco | 2.74 (1.50 – 5.02) | **0.001** | 2.17 (0.97 – 4.81) | | 0.057 |
| Marijuana use |  |  |  | |  |
| Never used marijuana | Reference | Reference | - | | - |
| Ever used marijuana | 2.05 (0.99 – 4.23) | **0.052** | - | | - |
| Illegal drugs use |  |  |  | |  |
| Never used illegal drugs | Reference | Reference | Reference | | Reference |
| Ever used illegal drugs | 2.69 (0.98 – 7.38) | **0.054** | 1.18 (0.35 – 3.96) | | 0.790 |
| Self-medicated for diagnosed conditions |  |  |  | |  |
| Not self-medicating | Reference | Reference | Reference | | Reference |
| Self-medicating | 1.32 (0.62 – 2.82) | 0.471 | 1.27 (0.57 – 2.86) | | 0.556 |
| Awareness of black market |  |  |  | |  |
| Not aware | Reference | Reference | - | | - |
| Aware | 1.11 (0.56 – 2.19) | 0.761 | - | | - |
