# Supplementary material for: Prevalence and correlates of prescription drug diversion and misuse among people living with HIV in the eThekwini district, KwaZulu-Natal, South Africa
Source: PLoS One. 2020 Dec 16;15(12):e0243718. doi: 10.1371/journal.pone.0243718 (PMC7744047; doi:10.1371/journal.pone.0243718)
Supplement: S3 Table — (DOCX) [file pone.0243718.s003.docx]

**S3 Table. Correlates of prescription drug misuse adjusted for geographical setting among PLWH in eThekwini district, Participants who ever misused prescription drugs, N = 89.** This is the S3 Table legend.

| **Variables** | **MODEL A** | | **MODEL B** | |
| --- | --- | --- | --- | --- |
|  | **Odds ratio (95% CI)** | ***p* value** | **Adjusted odds ratios (95% CI)** | ***p* value** |
| **Age in years** |  |  |  |  |
| 18 – 24 years | Reference | Reference | Reference | Reference |
| 25 – 29 years | 0.90 (0.33 – 2.46) | 0.839 | 0.78 (0.27 – 2.26) | 0.653 |
| 30 + years | 0.81 (0.33 – 2.00) | 0.645 | 0.77 (0.30 – 2.00) | 0.592 |
| **Gender** |  |  |  |  |
| Female | Reference | Reference | Reference | Reference |
| Male | 0.98 (0.59 – 1.62) | 0.941 | 0.86 (0.46 – 1.63) | 0.646 |
| **Diagnosed with other conditions** |  |  |  |  |
| Has other conditions | Reference | Reference | - | - |
| No other conditions | 1.14 (0.67 –1.92) | 0.632 | - | - |
| **Education** |  |  |  |  |
| Completed high school | Reference | Reference | - | - |
| Did not complete high school | 1.06 (0.65 –1.72) | 0.814 | - | - |
| **Employment status** |  |  |  |  |
| Employed | Reference | Reference | - | - |
| Unemployed | 1.16 (0.72 –1.86) | 0.548 | - | - |
| **Received income past month** |  |  |  |  |
| Received income | Reference | Reference | - | - |
| No income | 0.96 (0.54 –1.68) | 0.876 | - | - |
| **Homeless** |  |  |  |  |
| Never homeless past 30 days | Reference | Reference | Reference | Reference |
| Ever been homeless past 30 days | 2.25 (0.84 – 6.01) | 0.105 | 1.20 (0.33 – 4.35) | 0.775 |
| **Alcohol use** |  |  |  |  |
| Never used alcohol | Reference | Reference | - | - |
| Ever used alcohol | 0.84 (0.52 –1.36) | 0.489 | - | - |
| **Tobacco use** |  |  |  |  |
| Never used tobacco | Reference | Reference | Reference | Reference |
| Ever used tobacco | 1.31 (0.78 –2.20) | 0.301 | 1.22 (0.60 – 2.48) | 0.573 |
| **Marijuana use** |  |  |  |  |
| Never used marijuana | Reference | Reference | - | - |
| Ever used marijuana | 1.63 (0.87 –3.05) | 0.131 | - | - |
| **Illegal drugs use** |  |  |  |  |
| Never used illegal drugs | Reference | Reference | Reference | Reference |
| Ever used illegal drugs | 3.37 (1.37 – 8.30) | **0.008** | 2.18 (0.64 – 7.34) | 0.209 |
| **Self-medicated for diagnosed conditions** |  |  |  |  |
| Not self-medicating | Reference | Reference | Reference | Reference |
| Self-medicating | 2.91 (1.62 – 5.21) | **< 0.001** | 2.63 (1.44 – 4.82) | **0.002** |
| **Awareness of black market** |  |  |  |  |
| Not aware | Reference | Reference | Reference | Reference |
| Aware | 1.43 (0.84 – 2.43) | 0.191 | 1.19 (0.67 – 2.09) | 0.549 |
