# Supplementary material for: Prevalence and correlates of prescription drug diversion and misuse among people living with HIV in the eThekwini district, KwaZulu-Natal, South Africa
Source: PLoS One. 2020 Dec 16;15(12):e0243718. doi: 10.1371/journal.pone.0243718 (PMC7744047; doi:10.1371/journal.pone.0243718)
Supplement: S1 Questionnaire — (PDF) [file pone.0243718.s004.pdf]

Prescription drug diversion, misuse and abuse among people living with HIV in eThekwini District, KwaZulu-Natal

**Participant Study ID**	
**Date data collected**	
**Place/venue/recruitment site**	
**Start time**	
**End time**	

Introduction:

The questionnaire will assess your:

- Background
- Prescription medications
- Substance use
- Prescription drug diversion and non-diversion
- Prescription misuse and abuse
- Impressions of prescription medication black market
- ART adherence
- Stress, mental and emotional health
- Stigma

Instructions:

I, the interviewer will ask you questions. Please feel free to indicate when you are not comfortable with answering the presented question.

B. Background

B1. What is your gender?

Male 1
Female 2
Prefer not to answer 3

B2. What is your marital status?

Married 1
Single 2
Divorced 3
Widowed...4
Prefer not to answer 5

B3. How old were you on your last birthday?

			Age in years

B4. What population group do you consider yourself to be?

African 1
Asian/Indian 2
Coloured 3
White 4
Prefer not to answer 5
Other group 6
If other please specify: ....

B5. Do you have children (exclude children who have passed away)?

Yes	No
1	0

If No, Skip to B7

B6. If yes, how many children do you have (excluding children who have passed away? ....

B7. Did you complete high school?

Yes	No
1	0

If No, Then Skip to B9

B8. Have you completed any tertiary studies?

Yes	No
1	0

B9. What are you currently busy with?

Employed 1
Self-employed…………………………………………….2
Unemployed 3
Student…………………………………………………….4
Other.. ..5
If other Please specify ....

B10. Did you receive any income from any source in the last month?

Yes	No
1	0

B11. **Including yourself**, how many people depend on this income?

B12. What is the total monthly income of your household, including all sources, from all adult family members of the household? Please include jobs, any public assistance, unemployment benefits, disability, or other sources.

Less than R1000 per month 1
R1000 – R3000 per month 2
R3001 – R6000 per month 3
More than R6000 per month 4
I do not know... 5
Prefer not to answer.. 6

B13. What was your main source of income in the **last month**? **[Circle all that applies]**

a. Formal salary/earnings on which you pay income tax 1
b. Informal salary/earnings 2
c. Contributions by adult family members or relatives 3
d. Contributions by younger family members or relatives (<18 years) 4
e. Government pensions/Grants (e.g. old age pension, disability grant, child grant) 5
f. Grants/Donations by private welfare organizations 6
g. Other sources 7
If other sources please specify..

B14. How would you describe your household economic situation? **[Circle one that applies]**

Do not have enough money for basics such as food and clothes 1
Have enough money for basics but short for other things 2
Have enough money for most important things 3
Have some money, including for a few extras (e.g. Luxuries) 4
Prefer not to answer 5

B15. What type of neighbourhood do you live in? **[Circle all that applies]**

a. Orderly 1
b. Disorderly …………………………………………...2
c. Violent, crime-ridden.. 3
d. Drug-ridden... 4
e. Other 5

If other please specify ....

B16. In the past 30 days have you ever been homeless?

Yes	No
1	0

B17. Are you covered by a Medical Aid or any scheme that helps you pay private health care or drug services?

Yes	No
1	0

PM. Prescription Medication
**Read:** Now we would like to ask you about prescription medication. Prescription medication here refers to **any medication** (excluding traditional medicine). Please remember that everything you tell us is strictly confidential.

PM1. Beside been diagnosed with HIV, have you ever been diagnosed with any other condition or disease by a health care provider? ………

If, No Skip to question PM3,

Yes	No
1	0

PM2. What type of condition or disease were you diagnosed with? ………

Circle all that applies

a.	Tuberculosis……………….……………………………………………	1
b.	Hypertension……………………………………………………………	2
c.	Diabetes…………………………………………………………….	3
d.	Renal disease…………………………………………………………	4
e.	Asthma………………………………………………………………..	5
f.	Psychiatric ………………………………………………………….	6
g.	Other…………………………………………………………………..	7

If other please specify ……………………………………

PM3. Have you ever self-medicated yourself for the conditions or diseases that you were diagnosed with? ………

Yes	No
1	0

If Yes, specify the prescription medications

a. ______________________________

b. ______________________________

c. ______________________________

d. ______________________________

e. ______________________________

PM4. Have you ever been educated or trained on how to use or take any prescription medication? ……

Yes	No
1	0

PM5. Do you know of the Centralised Chronic Medicines Dispensing and Distribution (CCMDD), a program where by patients collect their medications at various pick-up points? ………

Yes	No
1	0

If No, then skip to PM7

PM6: Are you happy with the CCMDD program? ………

Yes	No
1	0

If **No,** specify the reason why you are not happy________________________________
____
____

PM7. Are you currently taking any prescription medication? ………

Yes	No
1	0

If No, then skip to the next section

PM8. For what condition or disease are you currently taking prescription medication for? ………

Circle all that applies

a.	HIV…………………………………………………..	1
b.	Tuberculosis (TB)………………………………………..	2
c.	Hypertension…………………. …………………….	3
d.	Diabetes……………………………………………….	4
e.	Renal disease………………………………………….	5
f.	Asthma……………………………………………….	6
g.	Psychiatric …………………………………….	7
h.	Other……………………………………………	8

If other please specify ……………………………………….

PM9. Currently, where do you often obtain or get your prescription medication? **Circle all that applies**

a.	Health care facility, prescribed during consultation…………………………	01
b.	Health care facility **without** a valid script ……………………………………	02
c.	Pharmacy **with** a valid script ………………..……………………………	03
d.	Pharmacy **without** a valid script ……………………………………..…….	04
e.	On the Internet **with** a valid script ………………………………….……………	05
f.	On the Internet **without** a valid script ……………. …………………………..	06
g.	Doctor shopping………………………………………………………………..	07
h.	Pharmacy shopping………………………………………………………....	08
i.	Family or friends…………………………………………………………….	09
j.	Pill broker or street vendors……………………………………………………	10
k.	Black market place………………………………….....................................	11

PM10. Are you currently enrolled in Centralised Chronic Medicines Dispensing and Distribution (CCMDD) program? ………

Yes	No
1	0

If **No,** specify the reason why not enrolled

____

____

If No, Skip to PM15

PM11. Have **you ever** collected your prescription medication **at any** pick-up point (exclude the health care facility where to do blood draws)?………

Yes	No
1	0

If No, Skip to PM15

PM12. Which pick-up point do you often use (exclude the health care facility where you do blood draws)? ……… **Circle all that applies**

a.	Pharmacy………………………………………………………………	1
b.	Clinic…………………………………………………………………	2
c.	Hospital………………………………………………………………	3
d.	Church………………………………………………………………	4
e.	School……………………………...	5
f.	Community hall………………………..	6
g.	Local store/supermarket……………………..	7
h.	Other…………………………………………………………………	8

If other please specify: ………………………………………………………

PM13. Have you ever collected your prescription medication at more than one pick-up point (exclude the health care facility where you do blood draws?

**Yes**	**No**
1	0

If No, skip to PM15

PM14. How often have you ever collected at more than one pick-up point (exclude the health care facility where you do blood draws?....

Once…………………………….......	1
Sometimes…………………………..	2
Always………..........................	3

PM15. Have you ever used prescription medication that was **not prescribed** to you by a health care provider?

**Yes**	**No**
1	0

If Yes, specify the prescription medications

a. ______________________________
b. ______________________________
c. ______________________________
d. ______________________________
e. ______________________________

S. Substance use								
**READ:** The following questions are about your use of alcohol and other drugs. Alcohol includes beer, wine, and hard liquor like in mixed drinks. "Other drugs" include: marijuana, ecstasy, cocaine, as well as any non-medical use of prescription-type drugs. When we talk about prescription drugs, we are NOT including when you use/used them as instructed under the direction of a health care provider, but instead when you used them to get high, for fun, to relax or to come down.								
**S1.** When was the **last time** that you used...?<br>[If '0' then SKIP to next substance.<br>If 1, 2, or 3 ask both **S2** and **S3**]<br>If 4, 5, go to question **S3.**	**Never**	**Within the past 7 days**	**Between 1-4 Weeks ago**	**Between 1-3 Months ago**	**Between 4-12 Months ago**	**More than 12 months ago**	**S2. During the past 90 days, on how many days have you used?**	**S3. How old were you the first time that you used?**
**1.** Alcohol including beer, wine and hard liquor	0	1	2	3	4	5	Days	Age
**2.** Marijuana (pot, weed, dagga, dope, grass, cannabis)	0	1	2	3	4	5	Days	Age
**3.** Tobacco as in cigarettes, cigars, or chewing tobacco	0	1	2	3	4	5	Days	Age
How many times **per day** do you usually smoke tobacco? Note: A pack is 20 cigarettes							Times	
**4.** Illegal drugs such as Heroin (smack, mud, china white brown, Mexican brown, brown sugar); Crack or freebase (smoked rock cocaine); Powdered cocaine (snorted or smoked), coke; Methamphetamine (Tik) or Crystal methamphetamine (Ice, meth, hawaian salt, crystal); Mandax (white buttons); Ecstasy (XTC, Adam, MDMA); Methamphetamine (Tik) or Crystal methamphetamine (Ice, meth, hawaian salt, crystal);); Whoonga (Nyaope)	0	1	2	3	4	5	Days	Age
If any illegal drug use, which ones did you use? ………………………………………………								

S. Substance use

READ: The following questions are about your use of alcohol and other drugs. Alcohol includes beer, wine, and hard liquor like in mixed drinks. "Other drugs" include: marijuana, ecstasy, cocaine, as well as any non-medical use of prescription-type drugs. When we talk about prescription drugs, we are NOT including when you use/used them as instructed under the direction of a health care provider, but instead when you used them to get high, for fun, to relax or to come down.

**S1.** When was the **last time** that you used...? [If ‘0’ then SKIP to next substance. If 1, 2, or 3 ask both **S2** and **S3**] If 4, 5, go to question **S3.**	**Never**	**Within the past 7 days**	**Between 1-4 Weeks ago**	**Between 1-3 Months ago**	**Between 4-12 Months ago**	**More than 12 months ago**	**S2. During the past 90 days, on how many days have you used?**	**S3. How old were you the first time that you used?**
**5.** Any sedatives (tranquilizers) such as Xanax, Valium, Ativan, Klonopin, Soma, Barbiturates or others NOT AS PRESCRIBED by a health care provider<br><br>**For euphoric effects such as excitement and happiness**	0	1	2	3	4	5	**Days**	**Age**
If any sedative use, which ones did you use? ……………………………………………….								
**6.** Any stimulants such as Adderall, Ritalin, Adipex or any other stimulants NOT AS PRESCRIBED by a health care provider<br><br>**For increasing the ability to concentrate, boost feelings and performance enhancing**	0	1	2	3	4	5	**Days**	**Age**
If any stimulant use, which ones did you use? …………………………………………………………….								
**7.** Any analgesics (painkillers) such as Opioids, Codeine, Tramadol, percocet, Vicodin, OxyContin, , Demerol, Darvon, Demerol, Morphine, Methadone, Fentanyl, Pentazocine, or others NOT AS PRESCRIBED by a health care provider<br><br>**For reducing pain or feeling high**	0	1	2	3	4	5	**Days**	**Age**
If any analgesics use, which ones did you use? **……………………………………………….**								

S. Substance use									
**READ:** The following questions are about your use of alcohol and other drugs. Alcohol includes beer, wine, and hard liquor like in mixed drinks. "Other drugs" include: marijuana, ecstasy, cocaine, as well as any non-medical use of prescription-type drugs. When we talk about prescription drugs, we are NOT including when you use/used them as instructed under the direction of a health care provider, but instead when you used them to get high, for fun, to relax or to come down.									
**S1.** When was the **last time** that you used...?<br><br>[If '0' then SKIP to next substance.<br>If 1, 2, or 3 ask both **S2** and **S3**]<br>If 4, 5, go to question **S3.**	**Never**	**Within the past 7 days**	**Between 1-4 Weeks ago**	**Between 1-3 Months ago**	**Between 4-12 Months ago**	**More than 12 months ago**	**S2. During the past 90 days, on how many days have you used?**	**S3. How old were you the first time that you used?**
**8.** Any antibiotics such as amoxicillin, ampicillin, cloxacillin, metronidazole, co-trimoxazole, ciprofloxacin, or others NOT AS PRESCRIBED by a health care provider	0	1	2	3	4	5	Days	Age
If any antibiotic use, which ones did you use?								
**9.** Any ARVS NOT AS PRESCRIBED by a health care provider	0	1	2	3	4	5	Days	Age
If any ARVs use, which ones did you use?								
**10.** Other drugs NOT AS PRESCRIBED by a health care provider	0	1	2	3	4	5	Days	Age
If any other, which ones did you use?								

S4. Have you ever attended AA (Alcoholics Anonymous), NA (Narcotics Anonymous), Cocaine Anonymous, or any other self-help group for your alcohol or drug use?

Yes	No
1	0

If No, Skip to S6

S5. During the past 90 days, on how many days have you attended AA (Alcoholics Anonymous), NA (Narcotics Anonymous), Cocaine or Crystal Meth Anonymous, or another self-help group for your alcohol or drug use?

Days	

S6. Is there any substance abuse history in your family

Yes	No
1	0

D. Prescription Drug Diversion

Read: The following questions are about selling, borrowing, sharing, giving away, doctor/pharmacy shopping, trading and stealing of prescription medications. Please remember that everything you tell us is **strictly confidential.**

D1. In the **past 90 days**, have you **bought** prescription medications from someone else or from a pharmacy **without a script** for your own use?

Yes	No
1	0

D2. Have you ever **diverted** (sold, borrowed, shared, gave-away, visited multiple doctors/pharmacies for the same medications or traded or stole) prescription medications?

Yes	No
1	0

If D2 No, then skip to next section

D3. Please specify the kind of diversion: **… [Circle all that apply]**

a. Sold……………………………………………………………1
b. Traded (exchange)………………………………………………2
c. Shared ……………………………………………………………3
d. Gave-away …………………………………………………………4
e. Visited multiple doctors/pharmacies for the same prescription medication……5
f. Borrowed ……………………………………………………………6
g. Stole ………………………………………………………………7

D4. When was the **last time**, that you diverted any prescription medications? **[Circle one]**

Within the past 7 days ………………………… 1
1-4 weeks ago ……………………………………… 2
1-3 months ago …………………………………… 3
4 to 12 months ago ……………………………… 4
More than 12 months ago ……………………… 5

D5. In total, on how many different times have you diverted prescription medications?

Times

D6. Please specify which prescription medications were diverted

V1: ________________________________

V2: ________________________________

V3: ________________________________

V4: ________________________________

V5: ________________________________

NOTE: SKIP to next section if you have not SOLD OR TRADED any prescription medication.

**D7.** From the previously mentioned diverted prescribed medications, when was **the last time** you sold/traded the prescription medications? **[If 0, then SKIP to next medication. If 1, 2, 3, ask both question 4 and 5.] If 4, 5 go to question 5.**	**Never**	**Within the past 7 days**	**Between 1-4 Weeks ago**	**Between 1-3 Months ago**	**Between 4-12 Months ago**	**More than 12 months ago**	**4.** During the past year, on how many different **TIMES** have you sold or traded…	**5.** Approximately how much money did you receive, on average, for one bottle (a month's supply)? Or if other, please describe ________
**Name of med**	**0**	**1**	**2**	**3**	**4**	**5**	**Times**	**Price**
**Name of med**	**0**	**1**	**2**	**3**	**4**	**5**	**Times**	**Price**
**Name of med**	**0**	**1**	**2**	**3**	**4**	**5**	**Times**	**Price**
**Name of med**	**0**	**1**	**2**	**3**	**4**	**5**	**Times**	**Price**
**Name of med**	**0**	**1**	**2**	**3**	**4**	**5**	**Times**	**Price**

**D8.**	When you sold your prescription medication was it to the same person or a different person?	Same person	Different person
V1.	………………………………………………………………..	1	0
V2.	………………………………………………………………..	1	0
V3.	………………………………………………………………..	1	0
V4.	………………………………………………………………..	1	0
V5.	………………………………………………………………..	1	0

If ARVs are one of the diverted prescribed medications then also answer D9.

D9. When was the **first time** that you sold or traded any of your prescribed HIV medications? | | | | | Year AND | | | Month

D10. What would you say were the **main reasons** that influenced your decision to sell your prescribed medications the **FIRST time**: **[DO NOT READ; Circle responses that best fit answers given]**

a. I needed the money for bills or living expenses ……………………………01
b. I needed the money for drugs or alcohol……………………………………02
c. I did not care……………………………………………………………...03
d. I gave up or I was hopeless /I wanted to die………………………………...04
e. I was not feeling sick………………………………………………………..05
f. My CD4 count or viral load numbers were good…………………………...06
g. It was leftover prescribed medications ……………………………………..07
h. I had an extra script for the same medicine…………………………………08
i. Other people around me were selling their medications as well…………...09
j. I thought I would be helping someone else………………………………....10
k. It was leftover prescribed medication that caused me side effects……….…11
l. Other reason…………………………………………………………………12

If other, please describe: ………..…………………………………………

D11. And when you **first** sold your prescribed medication, did **[Circle one]**

A stranger approached you about selling them 1
An acquaintance (someone you know) approached you about selling them 2
A good friend or a family member approached you about selling those 3
You went out to find someone to buy them from you 4
You became interested in selling your medications when you overheard others talking about it...……………………………………………………………………………………5

N. Non-Diversion

		Yes	No
**N1.**	Has anyone ever approached you about wanting to buy your prescription medications?	1	0

If No, Skip to next section

**N2.**	What was the **main reason** why you decided **not to sell** your prescription medications at the time you were approached?	Yes	No
a.	I need to take my medications to stay healthy	1	0
b.	The money was not worth it	1	0
c.	The hassle was not worth it	1	0
d.	I did not trust the person	1	0
e.	Concern about illegal nature of the transaction	1	0
f.	Some other reason	1	0
If other, please describe …………………………………………………………………….			

DM. Prescription Drug Misuse and Abuse

Read: We know that sometimes people do not follow instructions as prescribed by the health care provider. The following questions are about improper use of prescribed medications. **Please remember that everything you tell us is strictly confidential.**

**DM1. Prescription drug misuse:**<br>In the **past 90 days,** have you ever used prescription medications	Yes	No
a. Without a health care provider guidance or instructions	1	0
b. Non-medically (e.g. used it recreationally)	1	0
c. Without following the dosage instructions	1	0
d. Not following the correct way of administration of the medication	1	0
e. Not following the scheduled time periods	1	0
f. To further reduce the amount of pain you were feeling	1	0
g. To increase the ability to concentrate, boost feelings and performance enhancing	1	0
h. To experience pleasure or excitement and intense feelings of well-being and happiness	1	0
i. To get high	1	0

**DM2. Prescription drug abuse:**		
**a.** In the **past 90 days**, have you ever used prescription medications Intentionally to harm yourself	**Yes**	**No**
	1	0
**If yes,** please specify the type of prescription medication abused<br>……………………………………………………………………………………<br>……………………………………………………………………………………		
**If yes,** please give reasons why prescription medication was been abused<br>……………………………………………………………………………………<br>…………………………………………………………………………………….		
**b.** In the **past 90 days**, have you ever used prescription medications **more than** the prescribed period	**Yes**	**No**
	1	0
**If yes,** please specify the type of prescription medication used<br>……………………………………………………………………………………<br>……………………………………………………………………………………		

**DM3. Risks**:	Yes	No
Is misusing or abusing of prescription medication harmful your health?	1	0

I. Impressions of prescription medication black market

Read: I would like to get your general impression regarding trends in the street market for prescription medications

I1. How many people do you personally know who are involved in selling or trading prescription medications? _____________ People

I2. Are you aware of any prescription medication black market in eThekwini district

Yes	No
1	0

If No, skip to the next section

I3. Which places have you noticed black market in eThekwini district: [**Read all and Circle one response]**

a. Streets...1
b. Dealers 2
c. Unlicensed pharmacies 3
d. Taxi ranks 4
e. Other……………………………………………………..…5
If other please specify ....

AC. ART Adherence

AC1. **When** were you first diagnosed with HIV infection?

				AND			OR		

Year — Month — Years ago

AC2. **When** were you **first** prescribed to take any HIV medications (ARVs)?

				AND		

Year — Month

AC3. What was **your latest**, **most recent** viral load? [**Circle one]**

Undetectable 0
50 – 500 1
501 – 5 000 2
5 001 – 10 000 3
10 001 – 30 000 4
30 001 or more 5
Don't Know 6

AC4. **What** HIV medications (ARVs) are you currently prescribed?

V1 ____________________
V2. ____________________
V3. ____________________

AC5. How many pills are you prescribed to take each time?

	Anti-retroviral drug name	Number of times per day	Number of pills each time
**a.**			
**b.**			
**c.**			
**d.**			

AC6. How satisfied are you with your current treatment?

Very dissatisfied 1
Dissatisfied 2
Satisfied 3
Very satisfied 4

AC7. Has a health care provider ever told you that you were resistant to any HIV medication?

Yes	No
1	0

AC8. In the **past month**, have you experienced any side effects from your HIV medications such as diarrhea, vivid dreams, lack of sleep or any other?

Yes	No
1	0

AC9. Have **you ever** run out of any of your HIV medications before the refill was due (when you had both been taking them exactly as directed and had not given away or sold them)

Yes	No
1	0

AC10. When was the **last time** that you missed any of your HIV medications, even if it was just one pill or just one time? **[Circle one]**

Never .... 0 **[SKIP to Next Section]**
Within the past 7 days 1
1-4 weeks ago 2
1-3 months ago 3
4 to 12 months ago .... 4 → **[SKIP to Next Section]**
More than 12 months ago .... 5 → **[SKIP to Next Section]**

AC11. How many days in the **past month or 30 days**, have you missed taking any of your HIV medications, even if it was just one dose

___ ___ **(Days)**

AC12. How many doses did you miss?

	**Anti-retroviral drug name**	**Yesterday**	**In the past 2 to 7 days ago (not including yesterday)**
**a.**			
**b.**			
**c.**			
**d.**			
**e.**			

AC13. Some people find that they forget to take their pills on the weekend days. Did you miss any of your HIV medications **last weekend**, that is, last Saturday or Sunday

Yes	No
1	0

AC14. **Read:** Below are some reasons you may have missed taking any of your HIV medications.

In the past **90 days,** how often have you missed taking your medications because	**Never**	**Rarely**	**Sometimes**	**Often**	**Almost always**
**a.** You were away from home	0	1	2	3	4
**b.** You were too busy with responsibilities like taking care of children or going to appointments	0	1	2	3	4
**c.** You were getting high and did not remember	0	1	2	3	4
**d.** You were getting high and did not want to mix the medications with drugs	0	1	2	3	4
**e.** You were getting high and did not care	0	1	2	3	4
**f.** You simply forgot (not related to drug use)	0	1	2	3	4
**g.** You had difficulty in taking or swallowing pills wanted to avoid side effects	0	1	2	3	4
**h.** You wanted to avoid side effects	0	1	2	3	4
**i.** You did not want others to notice you were taking medications	0	1	2	3	4
**j.** You had a change in daily routine	0	1	2	3	4
**k.** You felt like the medications were harmful or toxic	0	1	2	3	4
**l.** You fell asleep or slept through a dose time	0	1	2	3	4
**m.** You felt sick or ill	0	1	2	3	4
**n.** You felt depressed or overwhelmed	0	1	2	3	4
**o.** You had problems taking pills at specified times (like with meals or on an empty stomach) or it conflicted with taking other medications	0	1	2	3	4
**p.** You felt good or healthy	0	1	2	3	4
**q.** You accidentally ran out of pills	0	1	2	3	4
**r.** You ran out of pills because you traded or sold them	0	1	2	3	4
**s.** You ran out of pills because they were stolen	0	1	2	3	4

ST. Stress, Mental and Emotional Health

Read: Many people experience difficulties in their lives from time to time. In the **past 90 days or 3 months**, have the following situations happened to you?

	Yes	No
**ST1.** Did you go without food, housing or other necessities because you didn't have the money?	1	0
**ST2.** Did the utility company threaten to cut off (or actually cut off) services such as electricity and water?	1	0
**ST3.** Did you get a divorce or break up with a partner	1	0
**ST4.** Did a family member or close friend become seriously ill	1	0
**ST5.** Did a family member or close friend die	1	0
**ST6.** Did you have trouble with your landlord	1	0

Read: the next questions are about common nerve, mental and psychological problems that many people experience. Please answer the next questions using yes or no.

During the past **12 months**, have you had **significant problems** with…	Yes	No
**ST7.** Feeling very trapped, lonely, sad, depressed or hopeless about the future?	1	0
**ST8.** Feeling easily annoyed, irritated or having trouble controlling your temper?	1	0
**ST9.** Feeling tired, having no energy or like you could not get things done?	1	0
**ST10.** Getting into a lot of arguments and feeling the urge to shout, throw things, beat, injure or harm someone?	1	0
**ST11.** Being unable or finding it difficult to control your worries?	1	0

ST12. When was the **last time (if ever)** you had any of those **significant** psychological problems that we talked about? **[Circle one]**

Never .... 0 **Skip to Next Section**
Within the past 7 days 1
1-4 weeks ago 2
1-3 months ago 3
4 to 12 months ago 4
More than 12 months ago 5

ST13. **During the past 90 days,** on how many days were you bothered those psychological problems?

Days

ST14. How **old** were you when you first started having these kinds of psychological problems?

Age

ST15. Has a health care provider ever told you that you have a mental, emotional or psychological problem?

Yes	No
1	0

If yes, please describe: **[DO NOT READ; Circle all that apply]**

a. Alcohol or drug dependence…………………………………………..1
b. Attention-deficit/hyperactivity disorder……………………………………2
c. Anxiety or phobia disorder………………………………………………3
d. Depression……………………………………………………………….4
e. Other ……………………………………………………………………….5
If other, please describe ____

ST16. Are you currently prescribed medication for mental, emotional, behavioural or psychological problems?

Yes	No
1	0

If yes, please describe the medication you are taking:

V1: ____

V2: ____

V3: ____

V4: ____

V5: ____

T. Stigma

T1. Are **family members** aware of your HIV status?

Yes	No
1	0

T2. Are people **outside** of your family aware of your HIV status?

Yes	No
1	0

T3. Has anyone ever made you use your own dishes and utensils or gave you plastic ones to use because you have HIV?

Yes	No
1	0

T4. Did family or friends ever cut down on visiting you at your home because you have HIV?

Yes	No
1	0

Please say Yes or No with these statements about having HIV and any stigma that you may or may not perceive	**Yes**	**No**
**T5.** Society looks down on people who have HIV	1	0
**T6.** People blame me for having HIV	1	0
**T7.** I am comfortable telling everyone I'm close to that I am living with HIV	1	0
**T8.** People think I am a bad person because I have HIV	1	0
**T9.** I am concerned if I go to the HIV clinic, someone I know might see me	1	0
**T10.** I feel abandoned by family members because I have HIV	1	0
**T11.** People avoid me because I have HIV	1	0
**T12.** People I am close to are afraid they will catch HIV from me	1	0
**T13.** I am concerned that if I am sick, people I know will find out about my HIV	1	0
**T14.** I feel ashamed to tell other people that I have HIV	1	0
**T15.** My family is comfortable talking about my HIV	1	0

T16. During the past 12 months, would you say your health in general was? **[READ and Circle one]**

Excellent 4
Very good 3
Good 2
Fair 1
Poor 0

Thank you! That is all the questions that we have for you today. We really appreciate your time and effort!
