# Supplementary material for: Prevalence and correlates of prescription drug diversion and misuse among people living with HIV in the eThekwini district, KwaZulu-Natal, South Africa
Source: PLoS One. 2020 Dec 16;15(12):e0243718. doi: 10.1371/journal.pone.0243718 (PMC7744047; doi:10.1371/journal.pone.0243718)
Supplement: Attachment — Submitted filename: PONE-D-19-31426_reviewer.pdf [file pone.0243718.s006.pdf]

PLOS ONE

Prevalence and Predictors of Prescription Drug Diversion, Misuse and Abuse among People Living with HIV in eThekwini District, KwaZulu-Natal, South Africa

--Manuscript Draft--

**Manuscript Number:**	PONE-D-19-31426
**Article Type:**	Research Article
**Full Title:**	Prevalence and Predictors of Prescription Drug Diversion, Misuse and Abuse among People Living with HIV in eThekwini District, KwaZulu-Natal, South Africa
**Short Title:**	Prevalence and Predictors of Prescription Drug Diversion, Misuse and Abuse among PLWH in eThekwini district
**Corresponding Author:**	Buyisile Chibi<br>University of KwaZulu-Natal College of Health Sciences<br>Durban, SOUTH AFRICA
**Keywords:**	medication diversion; drug diversion; drug misuse; medication misuse; Drug abuse; medication abuse; non-medical use
**Abstract:**	Background: Prescription drug diversion, misuse and abuse has increased over the past decade notably in high-income-countries and significantly contributing to the opioid epidemic. People living with HIV (PLWH) are particularly vulnerable to prescription drug diversion, misuse and abuse as mostly experience chronic pain, mental health problems and HIV-related illnesses. We investigated the prevalence and correlates of prescription drug diversion, misuse and abuse among PLWH in eThekwini district, KwaZulu-Natal.<br>Methods: A cross-sectional study was conducted among 392 PLWH, conveniently recruited from public health care facilities located in rural, semi-urban and urban areas of eThekwini district. Participants answered questions about their background; prescription medications; substance use and prescription drug diversion, misuse and abuse. Descriptive analysis was performed to estimate the prevalence of prescription drug diversion, misuse and abuse. Multivariable logistic regression was used to identify predictors of prescription drug diversion, and misuse.<br>Findings: Overall, 13% of participants reported lifetime prescription drug diversion. The most common type of diversion was using prescription medication not prescribed by a health care provider (11%), sharing of prescription medication (9%), buying prescription medication without a medical script (5%). Furthermore, 23% of participants reported prescription drug misuse in the past 90 days with using prescription medication without a health care providers' guidance (9%) and not following the scheduled time periods (8%) being the most common reported types of misuse. Prescription drug abuse was reported by 1% of PLWH. Substance use, homeless, no income, self-medicating and awareness of the black market were identified as risk factors for prescription drug diversion and misuse. There was no association between ART adherence and prescription drug diversion and misuse.<br>Conclusion: The study findings contribute to the limited data available on prescription drug diversion, misuse and abuse among PLWH in South Africa. The prevalence results underscore a need for urgent interventions when prescribing medications with potential risks. Addressing the risk of self-medicating is imperative for HIV care outcomes and to avert death.
**Order of Authors:**	Buyisile Chibi
	Nonhlanhla Yende-Zuma
	Tivani P Mashamba-Thompson
**Additional Information:**	
**Question**	**Response**
**Financial Disclosure**<br><br>Enter a financial disclosure statement that	Buyisile Chibi<br>Grant no. 107620<br>National Research Foundation

Powered by Editorial Manager® and ProduXion Manager® from Aries Systems Corporation

describes the sources of funding for the work included in this submission. Review the submission guidelines for detailed requirements. View published research articles from *PLOS ONE* for specific examples.<br><br>This statement is required for submission and **will appear in the published article** if the submission is accepted. Please make sure it is accurate.<br><br>**Unfunded studies**<br>Enter: *The author(s) received no specific funding for this work.*<br><br>**Funded studies**<br>Enter a statement with the following details:<br>• Initials of the authors who received each award<br>• Grant numbers awarded to each author<br>• The full name of each funder<br>• URL of each funder website<br>• Did the sponsors or funders play any role in the study design, data collection and analysis, decision to publish, or preparation of the manuscript?<br>• **NO** - Include this sentence at the end of your statement: *The funders had no role in study design, data collection and analysis, decision to publish, or preparation of the manuscript.*<br>• **YES** - Specify the role(s) played.<br><br>* typeset	Buyisile Chibi<br>Grant no. N/A<br>University of KwaZulu-Natal, College of Health Sciences<br><br>NO-the funders had no role in study design, data collection and analysis, decision to publish, or preparation of the manuscript.
**Competing Interests**<br><br>Use the instructions below to enter a competing interest statement for this submission. On behalf of all authors, disclose any competing interests that could be perceived to bias this work—acknowledging all financial support and any other relevant financial or non-financial competing interests.<br><br>This statement **will appear in the published article** if the submission is accepted. Please make sure it is accurate. View published research articles from *PLOS ONE* for specific examples.	NO- The authors have declared that no competing interests exist

Powered by Editorial Manager® and ProduXion Manager® from Aries Systems Corporation

**NO authors have competing interests**<br><br>Enter: *The authors have declared that no competing interests exist.*<br><br>**Authors with competing interests**<br><br>Enter competing interest details beginning with this statement:<br><br>*I have read the journal's policy and the authors of this manuscript have the following competing interests: [insert competing interests here]*<br><br>* typeset	
**Ethics Statement**<br><br>Enter an ethics statement for this submission. This statement is required if the study involved:<br><br>• Human participants<br>• Human specimens or tissue<br>• Vertebrate animals or cephalopods<br>• Vertebrate embryos or tissues<br>• Field research<br><br>Write "N/A" if the submission does not require an ethics statement.<br><br>General guidance is provided below. Consult the submission guidelines for detailed instructions. **Make sure that all information entered here is included in the Methods section of the manuscript.**	We commenced data collection process after obtaining the full ethical clearance from the University of KwaZulu-Natal's Biomedical Research Ethics Committee (Reference No. BE666/17), the KwaZulu-Natal Provincial Department of Health (Reference No. HRKM055/18, KZ_201802_026) and site clearance from all recruitment sites. Written consent was obtained from participants.

Powered by Editorial Manager® and ProduXion Manager® from Aries Systems Corporation

**Format for specific study types**<br><br>**Human Subject Research (involving human participants and/or tissue)**<br>• Give the name of the institutional review board or ethics committee that approved the study<br>• Include the approval number and/or a statement indicating approval of this research<br>• Indicate the form of consent obtained (written/oral) or the reason that consent was not obtained (e.g. the data were analyzed anonymously)<br><br>**Animal Research (involving vertebrate animals, embryos or tissues)**<br>• Provide the name of the Institutional Animal Care and Use Committee (IACUC) or other relevant ethics board that reviewed the study protocol, and indicate whether they approved this research or granted a formal waiver of ethical approval<br>• Include an approval number if one was obtained<br>• If the study involved *non-human primates*, add *additional details* about animal welfare and steps taken to ameliorate suffering<br>• If anesthesia, euthanasia, or any kind of animal sacrifice is part of the study, include briefly which substances and/or methods were applied<br><br>**Field Research**<br><br>Include the following details if this study involves the collection of plant, animal, or other materials from a natural setting:<br>• Field permit number<br>• Name of the institution or relevant body that granted permission	
**Data Availability**<br><br>Authors are required to make all data underlying the findings described fully available, without restriction, and from the time of publication. PLOS allows rare exceptions to address legal and ethical concerns. See the PLOS Data Policy and FAQ for detailed information.	Yes - all data are fully available without restriction

A Data Availability Statement describing where the data can be found is required at submission. Your answers to this question constitute the Data Availability Statement and **will be published in the article**, if accepted.<br><br>**Important:** Stating 'data available on request from the author' is not sufficient. If your data are only available upon request, select 'No' for the first question and explain your exceptional situation in the text box.<br><br>Do the authors confirm that all data underlying the findings described in their manuscript are fully available without restriction?	
**Describe where the data may be found in full sentences. If you are copying our sample text, replace any instances of XXX with the appropriate details.**<br><br>• If the data are **held or will be held in a public repository**, include URLs, accession numbers or DOIs. If this information will only be available after acceptance, indicate this by ticking the box below. For example: *All XXX files are available from the XXX database (accession number(s) XXX, XXX.*).<br>• If the data are all contained **within the manuscript and/or Supporting Information files**, enter the following: *All relevant data are within the manuscript and its Supporting Information files.*<br>• If neither of these applies but you are able to provide **details of access elsewhere**, with or without limitations, please do so. For example:<br><br>*Data cannot be shared publicly because of [XXX]. Data are available from the XXX Institutional Data Access / Ethics Committee (contact via XXX) for researchers who meet the criteria for access to confidential data.*<br><br>*The data underlying the results presented in the study are available from (include the name of the third party*	Data from this study are the property of the University of KwaZulu-Natal and can be made publicly available. All interested readers can access the data set from the author, Buyisile Chibi via this email address chibuyi@gmail.com and University of KwaZulu-Natal Biomedical Research Ethics Committee (BREC) from the following contacts: The Chairperson BIOMEDICAL RESEARCH ETHICS ADMINISTRATION Research Office, Westville Campus, Govan Mbeki Building University of KwaZulu-Natal P/Bag X54001, Durban, 4000 KwaZulu-Natal, South Africa Tel.: +27 31 260 4769 Fax: +27 31 260 4609 Email: BREC@ukzn.ac.za.

Powered by Editorial Manager® and ProduXion Manager® from Aries Systems Corporation

and contact information or URL).<br>• This text is appropriate if the data are owned by a third party and authors do not have permission to share the data.<br><br>* typeset	
Additional data availability information:	

Manuscript

Click here to access/download;Manuscript;Manuscript 15 Nov 2019.docx

Prevalence and Predictors of Prescription Drug Diversion, Misuse and Abuse among People Living with HIV in eThekwini District, KwaZulu-Natal, South Africa

Buyisile Chibi, *[1,2] Nonhlanhla Yende-Zuma [2] & Tivani P Mashamba-Thompson, [1]

[1]Discipline of Public Health Medicine, School of Nursing and Public Health, University of KwaZulu-Natal, Durban 4001, South Africa

[2] Centre for the AIDS Programme of Research in South Africa, 2nd Floor, Doris Duke Medical Research Institute, 719 Umbilo Road, Durban 4041, South Africa

***Correspondence:** Buyisile Chibi

[1]Discipline of Public Health Medicine, School of Nursing and Public Health, University of KwaZulu-Natal, Durban 4001, South Africa. E-mail: chibuyi@gmail.com

Co-author's email address:

Dr Tivani P. Mashamba-Thompson

Email address: Mashamba-Thompson@ukzn.ac.za

Dr Nonhlanhla Yende-Zuma

Email address: Nonhlanhla.Yende@caprisa.org

Abstract

Background: Prescription drug diversion, misuse and abuse has increased over the past decade notably in high-income-countries and significantly contributing to the opioid epidemic. People living with HIV (PLWH) are particularly vulnerable to prescription drug diversion, misuse and abuse as mostly experience chronic pain, mental health problems and HIV-related illnesses. We investigated the prevalence and correlates of prescription drug diversion, misuse and abuse among PLWH in eThekwini district, KwaZulu-Natal.

Methods: A cross-sectional study was conducted among 392 PLWH, conveniently recruited from public health care facilities located in rural, semi-urban and urban areas of eThekwini district. Participants answered questions about their background; prescription medications; substance use and prescription drug diversion, misuse and abuse. Descriptive analysis was performed to estimate the prevalence of prescription drug diversion, misuse and abuse. Multivariable logistic regression was used to identify predictors of prescription drug diversion, and misuse.

Findings: Overall, 13% of participants reported lifetime prescription drug diversion. The most common type of diversion was using prescription medication not prescribed by a health care provider (11%), sharing of prescription medication (9%), buying prescription medication without a medical script (5%). Furthermore, 23% of participants reported prescription drug misuse in the past 90 days with using prescription medication without a health care providers' guidance (9%) and not following the scheduled time periods (8%) being the most common reported types of misuse. Prescription drug abuse was reported by 1% of PLWH. Substance use, homeless, no income, self-medicating and awareness of the black market were identified as risk factors for prescription drug diversion and misuse. There was no association between ART adherence and prescription drug diversion and misuse.

Conclusion: The study findings contribute to the limited data available on prescription drug diversion, misuse and abuse among PLWH in South Africa. The prevalence results underscore a need for urgent interventions when prescribing medications with potential risks. Addressing the risk of self-medicating is imperative for HIV care outcomes and to avert death.

Keywords: drug diversion, drug misuse, drug abuse, non-medical use

Introduction

Prescription drug diversion, i.e. the illegal way of transferring controlled drugs from rightful owners to the black market for any use is a growing public health threat (1). In addition to this threat, prescription drug misuse, the improper use of medicines with or without a health care provider's guidance but clearly not compliant to instructions further increases the vulnerability (2). Meanwhile, prescription drug abuse, the non-medical use of drugs in harmful amounts is the main contributor to mortality rates due to drug overdose cases (3). The current world's battle is ending the AIDS epidemic globally (4). In 2018, about 37.9 million people were living with HIV globally with 1.7 million being new HIV infections and HIV related-deaths claimed 770 000 lives (5). Approximately, 23.3 million people living with HIV (PLWH) were receiving antiretroviral therapy however only

53% were virally suppressed (5). Since the universal test and treat, the 90-90-90 target as well as the UNIADS fast track strategy, only about 57% AIDS events and deaths reduction has been achieved with early ART initiation (6). From the global HIV prevalence, South Africa account for 19% (5) and KwaZulu-Natal province owning the highest HIV prevalence within the country (7). Barriers to achieving and maintaining viral suppression has been documented however prescription drug diversion, misuse and abuse might serve as one of the barriers (1, 8). One of the dangers of prescription drug diversion is transitioning into illicit drugs use therefore worsening the barriers to HIV care and challenging the HIV/AIDS targets to put a stop to the HIV epidemic. A recent scoping review study revealed that prescription drug diversion among PLWH came to existence as early as 1995 however there is lack of evidence in resource-limited-settings with high HIV epidemic (9).

PLWH often experience illnesses that correlates with prescription drug diversion, misuse and abuse. Evidence suggest that chronic pain the common comorbidity among PLWH correlates with prescription drug misuse (10, 11). Literature has also found substance use disorder or dependence as a risk factor for prescription drug diversion among PLWH (8, 10, 12-21). Prescription drug diversion, misuse and abuse has been associated with mental health problems such as depression symptoms, psychiatric disorder and anxiety as risk factors among PLWH (16, 18, 20, 22-24). Other factors associated with prescription drug diversion, misuse and abuse among PLWH in literature include male gender (16, 20), young in age (14, 22), HIV-related stigma (16), poor health (18), lower level of education (19, 24), unemployment or no income (25), past experience of diverting, misusing and abusing medicines (15, 26), in possession of addictive prescriptions (10, 24, 26) and living in high neighbourhood disorder (19). In the United States, these factors were reported being associated with new HIV infections (27). A recent scoping review study showed that there is limited research on prescription drug diversion in low-income-countries (28).

The trends of prescription drug diversion, misuse and abuse among PLWH are close to non-existence despite the emerging public health problem and the high HIV prevalence in the KwaZulu-Natal. The aim of this study was to investigate the prevalence and correlates of prescription drug diversion, misuse and abuse among PLWH in eThekwini district, KwaZulu-Natal. Furthermore, to investigate the association between ART adherence and prescription drug diversion, misuse and abuse. Knowing the extent and correlates associated with prescription drug diversion, misuse and abuse would enlighten health care providers and policy makers to incorporate appropriate and relevant interventions to address the problem when caring and managing HIV.

Methods

Study design

This study comprised of a cross-sectional survey conducted in eThekwini district, KwaZulu-Natal province, South Africa (Figure 1). We conducted the survey to investigate the extent of prescription drug diversion, misuse and abuse among PLWH HIV in eThekwini district. The study collected data from August 2018 to May 2019. We used an interviewer-administered questionnaire to collect data from a convenient sample of PLWH who at the time of survey were at the recruitment site.

Fig 1: The map of South Africa, zooming into KwaZulu-Natal highlighting districts and eThekwini district is one of the districts with high HIV prevalence.

Study setting

According to the eThekwini Municipality, the population size of eThekwini district was estimated at 3.7 million in 2016 of which about 1.9 million were PLWH (7). One of the key challenges related to eThekwini district health care service provision is the high prevalence of HIV/AIDS and also abuse of drugs and alcohol (29). However, until to date the extent of prescription drug diversion, misuse and abuse in eThekwini district is unknown.

Sampling method

We employed a stratified sampling method for sampling public health care clinics as recruitment sites for PLWH. We grouped all eThekwini district public health clinics providing HIV care programme into strata based on geographic settings i.e. rural, urban and semi-urban. Overall, eThekwini district has 118 public health care facilities of which 6% of the clinics were in rural areas, 53 % in urban areas and 41% clinics were in semi-urban areas. We applied a stratified randomization of public health clinics in the three strata. We did this to ensure that the obtained proportions of our sample matched those from all three geographical settings. We sought permissions to conduct the survey at each clinic through relevant authorities. We excluded clinics which were unable to give permission to participate in the study. We conveniently approached and invited study participants from the people who were waiting for healthcare workers consultations at the clinics. We employed outreach recruitment approach through Non-Governmental Organisation to reach people who were drug users and living with HIV.

Participants

We obtained written consent from 392 people who verbally confirmed to be living with HIV and aged 18 years and older. Some participants were assisted in their preferred language during consenting and conducting the survey. All participants were in eThekwini district at the time of the survey.

Data collection and instruments

We adapted the cross-sectional survey tool from the study conducted by Tsuyuki and Surratt (2015) (30), to suit our study population, setting and study aim. The study used hardcopies of the survey which consisted of ten categories i.e. background; prescription medications (PM); substance use; prescription drug diversion; non-diversion; prescription drug misuse and abuse; impressions of PM black market; antiretroviral therapy adherence; stress, mental and emotional health and stigma. Data was collected by the researcher and a trained research assistant. Prior to administering the questionnaire, we conducted a pilot test study among PLWH to ensure validity of the tool. The pilot test was used to improve precision, reliability, validity of data, identifying problems/omissions and time spent when completing the survey. The survey was conducted in English however questions were clarified to the participants in their preferred language. The survey lasted 60 minutes or less.

Ethical Considerations

We commenced data collection process after obtaining the full ethical clearance from the University of KwaZulu-Natal's Biomedical Research Ethics Committee (Reference No. BE666/17), the KwaZulu-Natal Provincial Department of Health (Reference No. HRKM055/18, KZ_201802_026) and site clearance from all recruitment sites. All questionnaires were identified by study ID (identity) numbers and not linked to consent forms.

Measures

This study focused on investigating the prevalence of prescription drug diversion, misuse and abuse among PLWH. We used the Gelberg–Andersen Behavioural Model conceptual framework to guide development the survey questions. The emphasises the importance of understanding how social vulnerability was associated with antiretroviral (ARV) diversion (31). This conceptual framework has been fully studied for health care utilization for vulnerable population (32). Our analysis examined three outcome measures. The first outcome is prescription drug diversion stem from the question "have you ever diverted (sold, borrowed, shared, gave-away, visited multiple doctors/pharmacies for the same medications or traded or stole) prescription medication (yes/no)?" The

second outcome is prescription drug misuse was recoded to “in the past 90 days have you ever misused prescription drugs (yes/no)?” Prescription drug misuse stem from several questions investigating different types of drug misuse such as “in the past 90 days, have you ever used prescription medication (PM) without a health care provider guidance or instructions?” The third and last, prescription drug abuse stems from the question “in the past 90 days, have you ever used PM intentionally to harm yourself (yes/no)? Additionally, self-reported data on ART adherence in the past 7 days stem from:

- How many pills (ARVs) are you prescribed to take each time?
    - Number of pills per day
    - Number of pills per time
- How many doses did you miss in the past 7 days?

Data management and analysis

We captured all data identified by study ID numbers into a password protected excel spreadsheet. The accuracy of source data was ensured by means of double capturing where the main author of this manuscript was the second data capturer. After data cleaning and verification, data was imported into R version 3.5.1 (2018-07-02)(33) for analysis. We stored all source documents in a secured place as back up.

The outcome variables were coded as follows: 1 = ever diverted prescription drugs vs. 0 = never diverted prescription drugs; 1 = misused prescription drugs in the last 90 days vs. 0 = never misused prescription drugs in the past 90 days; 1 = abused prescription drugs in the last 90 days vs. 0 = never abused prescription drugs in the past 90 days.

Continuous variables were summarised using means with standard deviation and compared across the two outcomes variables (diversion and misuse) using either independent samples t-test or Wilcoxon rank sum test. On the other hand, categorical variables were reported as frequencies with percentages and compared using Fisher’s exact test. The prevalence with its 95% confidence interval were calculated for prescription drug diversion, misuse and abuse among PLWH in eThekwini district.

We performed two types of multivariable logistic regression models to identify variables that were associated with diversion and misuse. For model A: we assessed the effect of each variable on the outcome (diversion or misuse)

controlling for geographical setting. For model B: we fitted all variables in the model including geographical setting to assess their collective effect on the outcome. Since we had too many variables to use in model B, we reduced them using the following strategy: we selected age and gender as these variables are known to be confounders of many exposure-outcomes relationships. Thereafter, and all variables that were plausible enough to be associated with each of the outcome variables were included in model B. This is a well thought and scientifically rigour strategy as opposed to using p-values to reduce the number of variables.

Therefore, the following variables were included in model B for prescription drug diversion outcome: age, gender, education, income, substance use (tobacco and illegal drugs) and self-medicating. Meanwhile, the variables included in model B for prescription drug misuse were age, gender, homeless, substance use (tobacco and illegal drugs), self-medicating and awareness of the black market.

Percentage ARV adherence was calculated by taking the total weekly ARV doses taken and dividing this number by the total ARV doses prescribed (and multiplying by 100 to attain a percentage from 0 to 100). Analysis were conducted to assess the association between percentage ARV adherence with prescription drug diversion or misuse or abuse.

Detailed analysis for prescription drug abuse was not performed since only few people reported prescription drug abuse in our study. All analyses were performed using R studio and Microsoft excel was used to plot graphs and forest plots.

Results

Characteristics of study participants

We received 102% response rate. Completed responses were received from 392 participants recruited from three geographic settings, i.e. rural setting 6% (n=25), urban setting 58% (n=226) and semi-urban setting 36% (n=141). The demographics of the 392 HIV positive participants were largely females 67% (n=263), average age 36.78 (± 10.0) years, African 99% (n=388), single 86% (n=335), and 4% (n=17) reported been homeless in the past 30 days (Table 1 and Table 2 in the supplementary material file). In our sample, 82% of the participants were on antiretroviral treatment with an average of 96% of ART adherence in the past 7 days. In our sample, 61% (n=238) of the participants have not completed high school. More than half of the sample were employed, 53% (n=209).

Rates of prescription drug diversion and its predictors

Overall, 13% (n=51) of the sample reported ever diverting prescription drugs (Figure 2). Majority, 11% (n=42), 9% (n=36) and 5% (n=20) reported using PM not prescribed by a health care provider, sharing PM and to have bought PM from someone else or from a pharmacy without a medical script for their own use in the past 90 days, respectively. The least type of diversions reported at 2% (n=9) or less were giving away, borrowing and trading of PM.

Fig 2: The prevalence of diversion and types of prescription drug diversion with 95% confidence interval reported by people living with HIV in eThekwini district

We determined correlations between characteristics of participants who ever diverted versus and never diverted prescription drugs (Table 1). In the multivariable model A (Figure 3): gender, income, substance use (tobacco, marijuana and illegal drugs) were significantly associated with prescription drug diversion. Males had 2-fold increased odds of diverting PM compared with females, [OR = 2.01 (95% CI: 1.10 – 3.65), $p = 0.022$]. Participants who had not received an income in the past month had 2-fold increased odds of diverting when compared with those who have received an income in the past month, [OR = 2.02 (95% CI: 1.08 – 3.80), $p = 0.029$]. Participants who used substances such as tobacco, marijuana and illegal drugs had almost 3-fold higher odds of diverting PM compared with non-substance users [OR = 2.74 (95% CI: 1.50 – 5.02), $p = 0.001$], [OR = 2.05 (95% CI: 0.99 – 4.23), $p = 0.052$] and [OR = 2.69 (95% CI: 0.98 – 7.38), $p = 0.054$], respectively.

Fig 3: Odds ratios showing the effect of each variable on diversion among PLWH in eThekwini district

Although found not to be statistically significant, participants with the following factors: not completed high school, unemployed, being homeless, used alcohol and self-medicating had more than 20% increased odds of diverting PM, [OR = 1.34 (95% CI: 0.72 – 2.49), $p = 0.359$], [OR = 1.23 (95% CI: 0.68 – 2.22), $p = 0.493$], [OR = 1.28 (95% CI: 0.35 – 4.60), $p = 0.710$], [OR = 1.35 (95% CI: 0.74 – 2.45), $p = 0.328$] and [OR = 1.32 (95% CI: 0.62 – 2.82), $p = 0.471$], respectively. Likewise, participants aged 30 years and older had more than 50% increased odds for diverting PM when compared to participants who were younger than 30 years old, [OR = 1.52 (95% CI: 0.51 – 4.53), $p = 0.449$].

In the multivariable model B participants with no income, ever used tobacco, ever used illegal drugs and self-medicated had increased odds of diverting PM, [OR = 1.86 (95% CI: 0.90 – 3.81), $p = 0.092$], [OR = 2.12 (95% CI: 0.95 – 4.70), $p = 0.065$], [OR = 1.31 (95% CI: 0.38 – 4.53), $p = 0.674$] and [OR = 1.22 (95% CI: 0.54 – 2.75), $p = 0.626$], respectively (Table 3 in the supplementary material file).

Rates of prescription drug misuse and its predictors

Overall, 23% (n=89) of the sample reported to have ever misused prescription drugs in the past 90 days (Figure 4). More than one third of the participants who misused drugs, 9% (n=35) and 8% (n=33) reported to have ever used PM without a health care provider guidance and not following the scheduled time periods in the past 90 days, respectively. Less than a quarter of the participants who misused drugs, 6% (n=25), 5% (n=18), 4% (n=14) reported to have used prescription drugs non-medically, to further reduce the amount of pain and without following the dosage instructions in the past 90 days, respectively. The least reported types of misuse, each reported at 1% (n=5) were not following the correct way of administration of the medication; to increase the ability to concentrate, boost feelings and performance enhancing; to experience pleasure and lastly to get high.

Fig 4: The prevalence of misuse and types of prescription drug misuse with 95% confidence interval reported by people living with HIV in eThekwini district

Comparisons were performed to compare characteristics of participants who ever misused versus never misused prescription drugs (Table 1). Participants who used illegal drugs had more than 3-fold higher odds of misusing PM [OR = 3.37 (95% CI: 1.37 – 8.30), $p = 0.008$] when compared to those who do not use illegal drugs. Moreover, participants who were self-medicating for the diagnosed conditions had almost 3-fold higher odds [OR = 2.91 (95% CI: 1.62 – 5.21), $p < 0.001$] for misusing PM (Figure 5). Furthermore, participants with the following characteristics: being homeless, tobacco use, marijuana use and aware of the black market had increased odds of misusing PM [OR = 2.25 (95% CI: 0.84 – 6.01), $p = 0.105$], [OR = 1.31 (95% CI: 0.78 – 2.20), $p = 0.301$, [OR = 1.63 (95% CI: 0.87 – 3.05), $p = 0.131$] and [OR = 1.43 (95% CI: 0.84 – 2.43), $p = 0.191$], respectively.

Fig 5: Odds ratios showing the effect of each variable on misuse among PLWH in eThekwini district

Results from model B showed that, self-medicating increased the odds of misusing PM [OR = 2.64 (95% CI: 1.44 – 4.84), *p* = 0.002]. In addition, participants with the following characteristics were associated with increased odds of misusing PM: being homeless, tobacco use, illegal drug use and aware of the black market, [OR = 1.14 (95% CI: 0.32 – 4.08), *p* = 0.842], [OR = 1.24 (95% CI: 0.61 – 2.51), *p* = 0.553], [OR = 2.13 (95% CI: 0.63 – 7.21), *p* = 0.222] and [OR = 1.21 (95% CI: 0.68 – 2.13), *p* = 0.516], respectively.

Rates of prescription drug abuse

Of all the sample, only 1% (n=5) reported to have ever used PM to intentionally harm themselves in the past 90 days. No regression analyses were performed for prescription drug abuse outcome since the proportion of participants who reported drug abuse was very small.

Table 1: Participants characteristics stratified by prescription drug diversion and misuse

Characteristics	Total N=392	Ever diverted prescription drugs, N=51	Did not divert prescription drugs, N=341	*p* value	Ever misused prescription drugs, N=89	Did not misuse prescription drugs, N=303	*p* value
Age in years, mean ±SD	36.8 ± 10.0	37.1 ± 8.5	36.7 ± 10.2	0.807	35.4 ± 8.6	37.2 ± 10.4	0.103
Age groups, n (%)							
18-24 years	38 (7)	4 (8)	34 (10)	0.172	9 (10)	29 (10)	0.607
25-29 years	83 (21)	6 (12)	77 (23)		22 (25)	61 (20)	
30 + years	271 (69)	41 (80)	230 (67)		58 (65)	213 (70)	
Gender, n (%)							
Male	129 (33)	24 (47)	105 (31)	**0.025**	29 (33)	100 (33)	1.000
Female	263 (67)	27 (53)	236 (69)		60 (67)	203 (67)	
Diagnosed with other conditions, n (%)	118 (30)	15 (29)	103 (30)	1.000	25 (28)	93 (31)	0.695
Education groups, n (%)							
Not completed high school	238 (61)	34 (67)	204 (60)	0.708	55 (62)	183 (60)	0.983
Completed only high school	114 (29)	13 (25)	101 (30)		25 (28)	89 (29)	
Completed both high school and tertiary studies	40 (10)	4 (8)	36 (10)		9 (10)	31 (26)	
Employment status, n (%)							
Employed	209 (53)	25 (49)	184 (54)	0.549	44 (49)	139 (46)	0.629
Unemployed	183 (47)	26 (51)	157 (46)		45 (51)	164 (54)	
Participants who received income past month, n (%)	300 (77)	33 (65)	267 (78)	0.065	69 (78)	231 (76)	0.113
Homeless, n (%)	17 (4)	3 (6)	15 (4)	0.543	7 (8)	11 (4)	0.144
Substance use, n (%)							
Ever used alcohol	186 (48)	27 (53)[a]	159 (47)	**0.032**	39 (44)	147 (49)	0.545
Ever used tobacco	106 (27)	24 (47)	83 (24)	**0.010**	28 (32)	78 (26)	0.342
Ever used marijuana	55 (14)	12 (24)	45 (13)	0.057	17 (19)	38 (13)	0.164
Ever used illegal drugs	21 (5)	6 (12)	15 (4)	0.065	10 (11)	11 (4)	**0.012**

Characteristics	Total N=392	Ever diverted prescription drugs, N=51	Did not divert prescription drugs, N=341	*p* value	Ever misused prescription drugs, N=89	Did not misuse prescription drugs, N=303	*p* value
Perceived misusing or abusing of prescription medication not harmful their health, n (%)	66 (17)[b]	8 (16)[c]	58 (17)[d]	0.495	13 (15)	53 (18)	0.725
Diagnosed with other conditions, n (%)	118 (30)	15 (29))	103 (30)	1.000	25 (28)	93 (31)	0.695
% ART adherence, mean ±SD	96.4 ± 12.9 [e]	94.5 ± 16.0 [f]	96.7 ± 12.4 [g]	0.366	96.0 ± 11.3 [h]	96.5 ± 13.36 [i]	0.705
Self-medicated for diagnosed conditions, n (%)	61 (16)	10 (20)	51 (15)	0.408	25 (28)	36 (12)	**< 0.001**
Awareness of black market, n (%)	94 (24)	13 (25)	81 (24)	0.861	26 (29)	68 (22)	0.203
Geographical setting, n (%)							
Rural	25 (6)	4 (8)	21 (6)		8 (9)	17 (6)	
Urban	226 (58)	35 (69)	191 (56)	0.132	55 (62)	171 (56)	0.218
Semi-urban	141 (36)	12 (24)	129 (38)		26 (29)	115 (38)	

[a] n=1:Missing data , [b] n=3: Not sure, [c] n=1: Not sure ,[d] n=2: Not sure, [e] n=12: Missing data, [f] n=1: Missing data, [g] n=11: Missing data, [h] n=4: Missing data, [i] n=8: Missing data

Association between ART adherence with prescription drug diversion, misuse

In average, our sample reported ART adherence level greater than 95% which is required in order to obtain successful ART outcome. Although, not found to be statistically significant participants who were diverting PM had lower ART adherence (96%) when compared with participants who never diverted PM (97%) in the past 7 days. Similarly, participants who were misusing PM had lower ART adherence (96%) when compared with participants who never misused PM (97%) in the past 7 days. No regression analyses were performed since the ART adherence of all groups were relatively similar.

Discussion

We sought to investigate the prevalence and correlates of prescription drug diversion, misuse and abuse in a cohort of HIV-infected adults in eThekwini district, KwaZulu-Natal province. In this study, the overall prevalence of prescription drug diversion among PLWH was found to be 13% during their lifetime meanwhile 23% of participants reported prescription drug misuse in the past 90 days and only a few participants reported prescription drug abuse. Self-medicating was the only predictor found to be statistically significant for predicting prescription drug misuse among PLWH.

Although there is limited evidence on prescription drug diversion in resource limited settings, numerous studies have been conducted in the United States where higher rates of prescription drug diversion have been reported among PLWH when compared to our findings (9). For example, a clinical trial conducted in the United States among men who have sex with men living with HIV on treatment documented that 27% of the participants reported ever sold their treatment and 19% had diverted their treatment in the past year (17). Prescription drug misuse is a silent growing problem globally however literature has documented a wide prevalence spectrum. Nonetheless, our prevalence of prescription drug misuse is within the reported spectrum. For instance, two longitudinal studies conducted in the United States among PLWH reported 21% of opioid misuse in the past 90 days (34) and 37% of abnormal use of prescription opioids in the past 90 days (35). Yet other studies in the United States reported lower prevalence when compared to our findings. For example, 19% of history of prescription drug misuse was reported among HIV-infected individuals in the US military (36). Meanwhile a cross-sectional study in the United States among PLWH reported 11% of non-medical use of prescription opioids in the past year (37).

Interestingly, the prevalence of prescription drug diversion and misuse among PLWH is higher when compared with the general population. In support to this, according to the findings of the South African Demographic and Health Survey conducted in 2016 about 2% of the participants aged 15 years and older reported to have consumed codeine containing products for non-medical reasons (38). Furthermore, a recent study in South Africa reported 6% of non-medical use of codeine containing mixtures among the general population (39). Meanwhile findings from the 2013 National Survey on Drug Use and Health documented 7% of prescription drug misuse among adults (40) while current trend analysis reports lower prevalence in 2018 when compared with rates reported in 2015-2017 among people aged 12 years or older (41). Similar to our study findings, several studies conducted in upper-middle-income countries such as Jordan(42-44) , China(45, 46), Iran(47) and Turkey(48) had shown that prescription drug misuse cases such as to stop taking medications earlier than instructed (42, 45), accessing of prescription medicines without a doctor's medical script (43-47), using medication without a medical indication (48) and taking higher dosages in the event of missed dose (42) were also reported among the general population. Though, only a few PLWH reported prescription drug abuse, increases in prescription drug overdose deaths have coincided with an increase rates of drug diversion, drug misuse and prescription drug-related illnesses (49-51). Despite that there was no association between ART adherence and prescription drug diversion and misuse in the past 7 days, earlier research in the United States found that prescription opioids misuse was linked with less than 90% ART adherence (52). Similarly, a study conducted in 2017 in the United States reported that opioid use was significantly associated with failure to achieve viral load suppression (53).

We observed that after adjusting for geographical settings, PLWH who were aged 30 years and older, male, had no income and used substances were at increased risk of diverting prescription drugs. Meanwhile, PLWH who used substances, self-medicated themselves and aware of the black market had increased risk of misusing prescription drugs. Similar to our findings, substance use disorder or dependence (8, 10, 12-21), male gender (16, 20) and unemployment or no income (25) has been reported as risk factors for prescription drug diversion among PLWH. Moreover, evidence has shown that men access health care services including HIV treatment services later at their illness stage than women (54). Nevertheless, our findings highlight a hidden behaviour of diversion that might be practiced by most men living with HIV when they are in need to medicines. Though literature has reported females as more likely to misuse prescription drugs than males (55). This would mean that females frequently access health care services and get prescribed medicines, but clearly they do not follow the instructions of taking the medicines. It is imperative to design tailor-made interventions specific for males and females for

different age groups to mitigate the barriers to optimal health care outcomes and prevent potential risks. Though self-medicating is a commonly practiced behaviour, inappropriate self-medication practices has potential risks but not limited to incorrect manner of drug administration, incorrect dosage consumption, incorrect choice of medicines, drug adverse reactions, risky drug-drug interactions and risk of developing drug dependence and abuse (56). This strongly support the association between misuse and self-medicating. Inappropriate self-medicating might hinder progress to achieve and maintain viral suppression outcome among PLWH. We recommend additional awareness interventions to supplement the medicine information pamphlet that is only read and comprehended by only few consumers. PLWH often presents with known risk factors associated with prescription drug diversion, misuse and abuse of which includes but not limited to substance use, mental health problems and chronic pain. They frequently get prescribed controlled medications to alleviate the experienced health problems, therefore consideration of the correlates of prescription drug diversion, misuse and abuse among PLWH is critical.

Strengths and limitations

Despite our limitations, this study is among the first to investigate prescription drug diversion, misuse and abuse among PLWH in eThekwini district. Although the study consisted of sensitive questions, our study provides valuable information on prescription drug diversion, misuse and abuse. Behaviour practices related questions were based on self-report and these may be implicated by recall and social desirability bias. The exclusion of metal health variable in the analysis could be a limitation since mental health problems is one on the risk factors. Gaps were identified in the study questionnaire and the questionnaire did not adequately cater for the resource limited setting like eThekwini district since most people had low knowledge and awareness of different types of prescription drugs. Comparisons were limited since the prevalence of prescription drug diversion, misuse and abuse were investigated in different time periods in our study and in literature. Laboratory HIV viral load test results can be used to support the self-reported ART adherence. Our findings are generalizable only to PLWH from resource-limited settings accessing care in public health care facilities, whereas prescription drug diversion, misuse and abuse among PLWH accessing care in private facilities might be different.

Conclusion

The high prevalence rate of prescription drug diversion and misuse among PLWH underscore the need of integrated surveillance tools at all levels in the health care system. Monitoring the dispensing of medicine and evaluation of medicine usage by health care providers is highly recommended for routine prescriptions and for

patients presenting with risk factors. We recommend educational and awareness campaigns directed at increasing patient or public knowledge of the potential risks and promoting a greater awareness among health care providers and to the public. Caution should be taken when prescribing of medicines with potential risks for diversion, misuse and abuse especially when the patient is presenting with risk factors. This study estimated the prevalence of prescription drug diversion, misuse and abuse among PLWH in eThekwini district. Prescription drug diversion, misuse and abuse practice needs urgent attention since there is a risk of transitioning to illegal drug use.

Declarations

Ethics approval and consent to participate

The University of KwaZulu-Natal's Biomedical Research Ethics Committee (Reference No. BE666/17), the KwaZulu-Natal Provincial Department of Health (Reference No. HRKM055/18, KZ_201802_026) and site clearance from all recruitment sites were obtained prior to data collection. All participants consented before data collection resumed.

Consent for publication

Not applicable

Availability of data and material

Data from this study are the property of the University of KwaZulu-Natal and can be made publicly available. All interested readers can access the data set from the author, Buyisile Chibi via this email address chibuyi@gmail.com and University of KwaZulu-Natal Biomedical Research Ethics Committee (BREC) from the following contacts: The Chairperson BIOMEDICAL RESEARCH ETHICS ADMINISTRATION Research Office, Westville Campus, Govan Mbeki Building University of KwaZulu-Natal P/Bag X54001, Durban, 4000 KwaZulu-Natal, South Africa Tel.: +27 31 260 4769 Fax: +27 31 260 4609 Email: BREC@ukzn.ac.za.

Competing interests

None declared

Funding

Financial source: University of KwaZulu-Natal, College of Health Sciences

Role: None

Funds used for: Data collection, study materials and recourses

Financial source: National Research Foundation

Role: None

Funds used for: Data collection, study materials and recourses

The corresponding author had full access to all the data in the study and had final responsibility for the decision to submit for publication.

Authors' contributions

BC and TPM-T conceptualized and designed the study. BC conducted literature search, data collection, data analysis, presentation of figures and tables, results interpretation, prepared the first manuscript draft prepared all drafts. NY-Z, verified all analysis, presentation of figures and tables, results interpretation and reviewed the all drafts. TPM-T, interpreted the results and reviewed all drafts and approved the final manuscript.

Acknowledgements

The authors wish to acknowledge the participation of the following institutions/ organisations: Human Sciences Research Council, KwaZulu-Natal Department of Health, TB HIV Care Organisation, as well all study participants and research assistants.

References

1. Inciardi JA, Surratt HL, Kurtz SP, Burke JJ. The diversion of prescription drugs by health care workers in Cincinnati, Ohio. Subst Use Misuse. 2006;41(2):255-64.

2. Casati A, Sedefov R, Pfeiffer-Gerschel T. Misuse of medicines in the European Union: a systematic review of the literature. Eur Addict Res. 2012;18(5):228-45.

3. Akerele E, Olupona T. Drugs of Abuse. Psychiatr Clin North Am. 2017;40(3):501-17.

4. Assembly UN General. Political Declaration on HIV and AIDS: on the fast-track to accelerate the fight against HIV and to end the AIDS epidemic by 2030. New York: United Nations; 2016.

5. UNAIDS. Global AIDS update 2019: Communities at the centre [Accessed date: 11 October 2019]. Available from: https://www.unaids.org/en/resources/documents/2019/2019-global-AIDS-update.

6. Abdool Karim SS. Overcoming impediments to global implementation of early antiretroviral therapy. Mass Medical Soc; 2015.

7. Karim QA, Karim SSA, Frohlich JA, Grobler AC, Baxter C, Mansoor LE, et al. Effectiveness and safety of tenofovir gel, an antiretroviral microbicide, for the prevention of HIV infection in women. Science. 2010;329(5996):1168-74.

8. Inciardi JA, Surratt HL, Kurtz SP, Cicero TJ. Mechanisms of prescription drug diversion among drug-involved club- and street-based populations. Pain Med. 2007;8(2):171-83.

9. Chibi B, Torres NF, Sokhela ZP, Mashamba-Thompson TP. Prescription drug diversion among people living with HIV: a systematic scoping review. Public Health. 2019;177:26-43.

10. Vijayaraghavan M, Penko J, Bangsberg DR, Miaskowski C, Kushel MB. Opioid analgesic misuse in a community-based cohort of HIV-infected indigent adults. JAMA Intern Med. 2013;173(3):235-7.

11. Tsao JC, Plankey MW, Young MA. Pain, psychological symptoms and prescription drug misuse in HIV: A literature review. J Pain Manag. 2012;5(2):111-8.

12. Tsao JCI, Stein JA, Dobalian A. Pain, problem drug use history, and aberrant analgesic use behaviors in persons living with HIV. Pain. 2007;133(1-3):128-37.

13. Tsao JC, Stein JA, Dobalian A. Sex differences in pain and misuse of prescription analgesics among persons with HIV. Pain Med. 2010;11(6):815-24.

14. Vijayaraghavan M, Penko J, Guzman D, Miaskowski C, Kushel MB. Primary care providers' judgments of opioid analgesic misuse in a community-based cohort of HIV-infected indigent adults. J Gen Intern Med. 2011;26(4):412-8.

15. Genberg BL, Gillespie M, Schuster CR, Johanson CE, Astemborski J, Kirk GD, et al. Prevalence and correlates of street-obtained buprenorphine use among current and former injectors in Baltimore, Maryland. Addictive behaviors. 2013;38(12):2868-73.

16. Surratt HL, Kurtz SP, Cicero TJ, O'Grady C, Levi-Minzi MA. Antiretroviral medication diversion among HIV-positive substance abusers in South Florida. American journal of public health. 2013;103(6):1026-8.

17. Kurtz SP, Buttram ME, Surratt HL. Vulnerable infected populations and street markets for ARVs: Potential implications for PrEP rollout in the USA. AIDS Care. 2014;26(4):411-5.

18. Newville H, Roley J, Sorensen JL. Prescription medication misuse among HIV-infected individuals taking antiretroviral therapy. Journal of substance abuse treatment. 2015;48(1):56-61.

19. Surratt HL, Kurtz SP, Levi-Minzi MA, Chen M. Environmental Influences on HIV Medication Adherence: The Role of Neighborhood Disorder. American journal of public health. 2015;105(8):1660-6.

20. Tsuyuki K, Surratt HL, Levi-Minzi MA, O'Grady CL, Kurtz SP. The Demand for Antiretroviral Drugs in the Illicit Marketplace: Implications for HIV Disease Management Among Vulnerable Populations. AIDS Behav. 2015;19(5):857-68.

21. Robinson-Papp J, Elliott K, Simpson DM, Morgello S. Problematic prescription opioid use in an HIV-infected cohort: the importance of universal toxicology testing. J Acquir Immune Defic Syndr. 2012;61(2):187-93.

22. Roux P, Carrieri PM, Cohen J, Ravaux I, Spire B, Gossop M, et al. Non-medical use of opioids among HIV-infected opioid dependent individuals on opioid maintenance treatment: the need for a more comprehensive approach. Harm reduction journal. 2011;8:31.

23. Surratt HL, O'Grady CL, Levi-Minzi MA, Kurtz SP. Medication adherence challenges among HIV positive substance abusers: the role of food and housing insecurity. AIDS Care. 2015;27(3):307-14.

24. Wixson SE. Medication misadventures: the case of benzodiazepines: University of Kentucky; 2015.

25. O’Grady CL, Kurtz SP, Surratt HL. Antiretroviral medication diversion in South Florida: Prescription types and motivations. Drug & Alcohol Dependence. 2014;140:e164-e.

26. Vijayaraghavan M, Freitas D, Bangsberg DR, Miaskowski C, Kushel MB. Non-medical use of non-opioid psychotherapeutic medications in a community-based cohort of HIV-infected indigent adults. Drug Alcohol Depend. 2014;143:263-7.

27. Prejean J, Song R, Hernandez A, Ziebell R, Green T, Walker F, et al. Estimated HIV incidence in the United States, 2006-2009. PloS one. 2011;6(8):e17502.

28. Chibi B, Torres NF, Sokhela ZP, Mashamba-Thompson TP. Risk factors for prescription drug diversion among people living with HIV: a systematic scoping review. Int J Evid Based Healthc. 2019.

29. Gumede-Moyo S, Filteau S, Munthali T, Todd J, Musonda P. Implementation effectiveness of revised (post-2010) World Health Organization guidelines on prevention of mother-to-child transmission of HIV using routinely collected data in sub-Saharan Africa: a systematic literature review. Medicine. 2017;96(40):e8055. doi: 10.1097/MD.0000000000008055.

30. Tsuyuki K, Surratt HL. Antiretroviral drug diversion links social vulnerability to poor medication adherence in substance abusing populations. AIDS Behav. 2015;19(5):869-81.

31. Tsuyuki K, Surratt HL. Antiretroviral Drug Diversion Links Social Vulnerability to Poor Medication Adherence in Substance Abusing Populations. AIDS Behav. 2015;19(5):869-81.

32. Gelberg L, Andersen RM, Leake BD. The Behavioral Model for Vulnerable Populations: application to medical care use and outcomes for homeless people. Health Serv Res. 2000;34(6):1273-302.

33. Venables W, Smith D. An Introduction to R Notes on R: A Programming Environment for Data Analysis and Graphics Version 3.5. 1 (2018-07-02). R. Gentleman & R. Ihaka Copyright c. 1997.

34. Jeevanjee S, Penko J, Guzman D, Miaskowski C, Bangsberg DR, Kushel MB. Opioid analgesic misuse is associated with incomplete antiretroviral adherence in a cohort of HIV-infected indigent adults in San Francisco. AIDS and behavior. 2014;18(7):1352-8.

35. Hansen L, Penko J, Guzman D, Bangsberg DR, Miaskowski C, Kushel MB. Aberrant Behaviors With Prescription Opioids and Problem Drug Use History In a Community-Based Cohort of HIV-Infected Individuals. J Pain Symptom Manage. 2011;42(6):893-902.

36. Deiss R, Byrne M, Larson D, Ganesan A, Noiman A, Garges E, et al. Prescription Drug Misuse in an HIV-Infected US Military Cohort. Open Forum Infectious Diseases. 2018;5.

37. Turner AN, Maierhofer C, Funderburg NT, Snyder B, Small K, Clark J, et al. High levels of self-reported prescription opioid use by HIV-positive individuals. AIDS care. 2016;28(12):1559-65.

38. National Department of Health (NDoH) SSASS, South African Medical Research Council (SAMRC), and ICF. South Africa Demographic and Health Survey 2016. Pretoria, South Africa, and Rockville, Maryland, USA: NDoH, Stats SA, SAMRC, and ICF.; 2019.

39. Wells JS, Bergin M, Van Hout MC, McGuinness P, De Pleissisc J, Rich E, et al. Purchasing Over The Counter (OTC) Medicinal Products Containing Codeine - Easy Access, Advertising, Misuse and Perceptions of Medicinal Risk. J Pharm Pharm Sci. 2018;21(1):30049.

40. Ford J, Wright L. Prescription Drug Misuse and Arrest History. Subst Use Misuse. 2017;52(13):1772-7.

41. Substance Abuse and Mental Health Services Administration. Key substance use and mental health indicators in the United States: Results from the 2018 National Survey on Drug Use and Health (HHS Publication No. PEP19-5068, NSDUH Series H-54). 2019.

42. Haddad M, Ebada ME. Demographic and socioeconomic characteristics of outpatients could modify their attitude towards misusing medications in northern Jordan. J Public Health Res. 2017;6(1):24-30.

43. Yaylaci S, Tosun O, Sahin O, Genc AB, Aydin E, Demiral G, et al. Misuse of levothyroxine and the rate of achieving target thyroid-stimulating hormone in levothyroxine treatment. Biomed Res. 2017;28(6):2661-5.

44. Wazaify M, Bdair A, Al-Hadidi K, Scott J. Doping in Gymnasiums in Amman: The other side of Prescription and Nonprescription Drug Abuse. Substance Use & Misuse. 2014;49(10):1296-302.

45. Wang X, Peng D, Wang W, Xu Y, Zhou X, Hesketh T. Massive misuse of antibiotics by university students in all regions of China: implications for national policy. Int J Antimicrob Agents. 2017;50(3):441-6.

46. Peng D, Wang X, Xu Y, Sun C, Zhou X. Antibiotic misuse among university students in developed and less developed regions of China: a cross-sectional survey. Glob Health Action. 2018;11(1):1-7.

47. Shirzadeh E, Shomoossi N, Abdolahzadeh H. Topical Anesthetic Misuse in Patients Admitted to Sabzevar Eye Clinic in Iran. Acta Facultatis Medicae Naissensis. 2016;33(4):287-94.

48. Bulbul F, Alpak G, Unal A, Tahsin Kilic OH, Ermis B, Savas HA. Misuse of Methylphenidate and Attitudes About Methylphenidate Among Resident Physicians. Uzmanlık eğitimi gören hekimlerin metilfenidatı uygunsuz kullanımı ve metilfenidata yaklaşımları. 2014;24(2):135-8.

49. CDC grand rounds: prescription drug overdoses - a U.S. epidemic. MMWR Morb Mortal Wkly Rep. 2012;61(1):10-3.

50. Vital signs: overdoses of prescription opioid pain relievers---United States, 1999--2008. MMWR Morb Mortal Wkly Rep. 2011;60(43):1487-92.

51. Okie S. A flood of opioids, a rising tide of deaths. N Engl J Med. 2010;363(21):1981-5.

52. Jeevanjee S, Penko J, Guzman D, Miaskowski C, Bangsberg DR, Kushel MB. Opioid analgesic misuse is associated with incomplete antiretroviral adherence in a cohort of HIV-infected indigent adults in San Francisco. AIDS and behavior. 2014;18(7):1352-8.

53. Flores J, Liang Y, Ketchum NS, Turner BJ, Bullock D, Villarreal R, et al. Prescription Opioid Use is Associated with Virologic Failure in People Living with HIV. AIDS Behav. 2017.

54. Mills EJ, Beyrer C, Birungi J, Dybul MR. Engaging men in prevention and care for HIV/AIDS in Africa. PLoS Med. 2012;9(2):e1001167.

55. Cheah YK, Chong YW. Factors influencing medication label viewing in Malaysia. Glob Health Promot. 2018;25(1):63-72.

56. Ruiz ME. Risks of self-medication practices. Curr Drug Saf. 2010;5(4):315-23.

Figure 1

Click here to access/download;Figure;Fig 1.tif

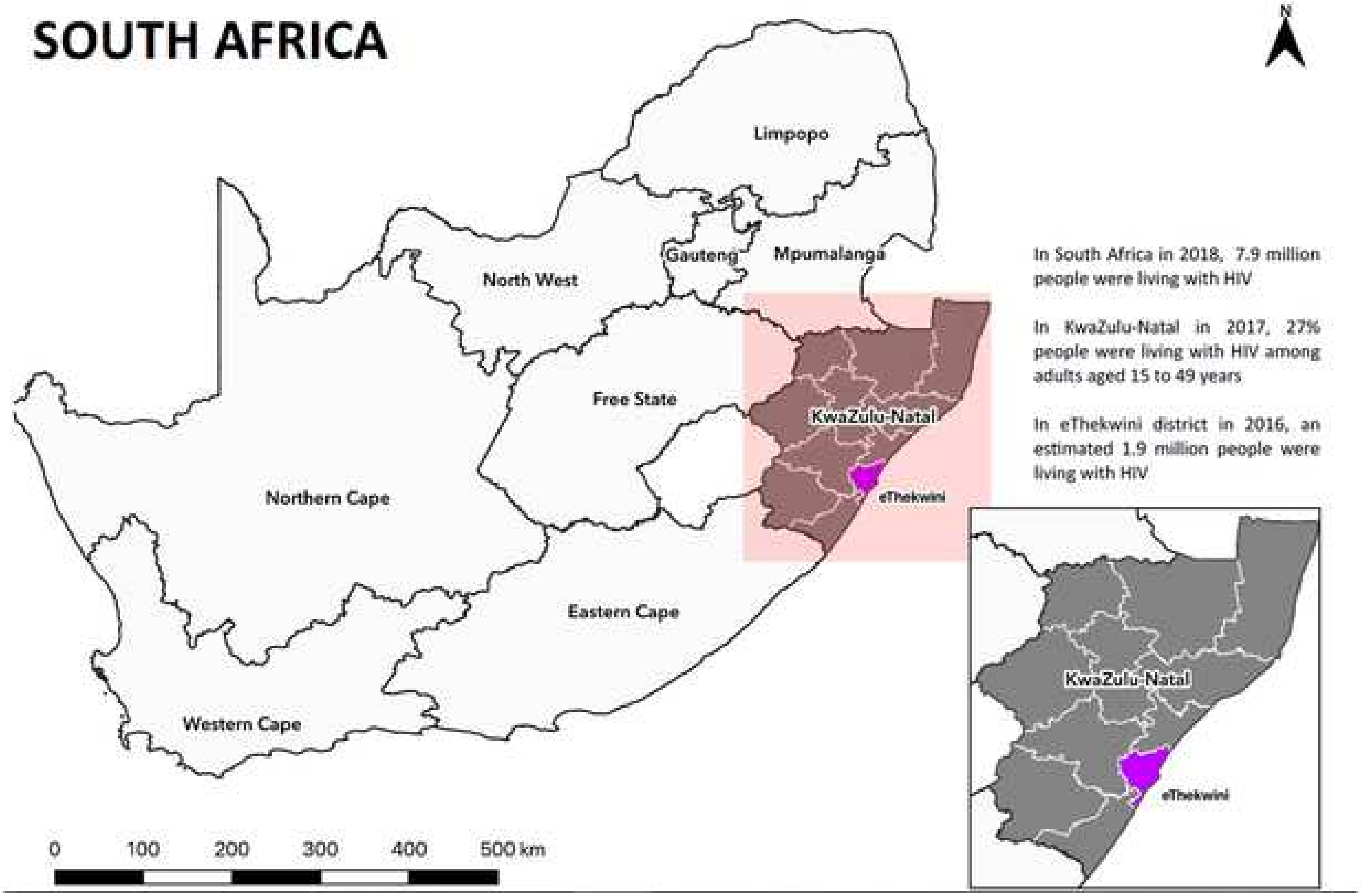

Figure 2

Click here to access/download;Figure;Fig 2.tif

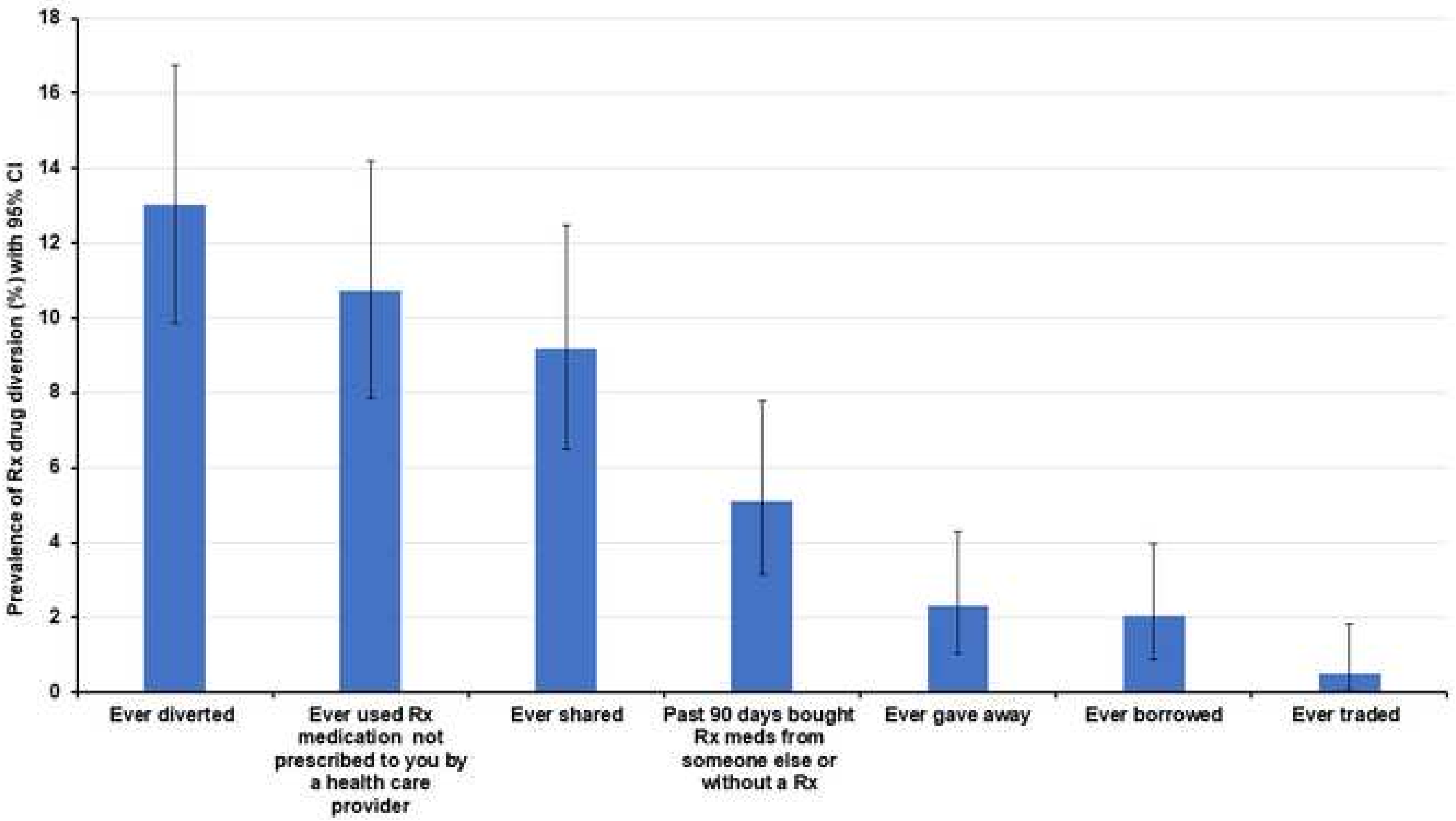

Figure 3 Click here to access/download;Figure;Fig 3.TIF

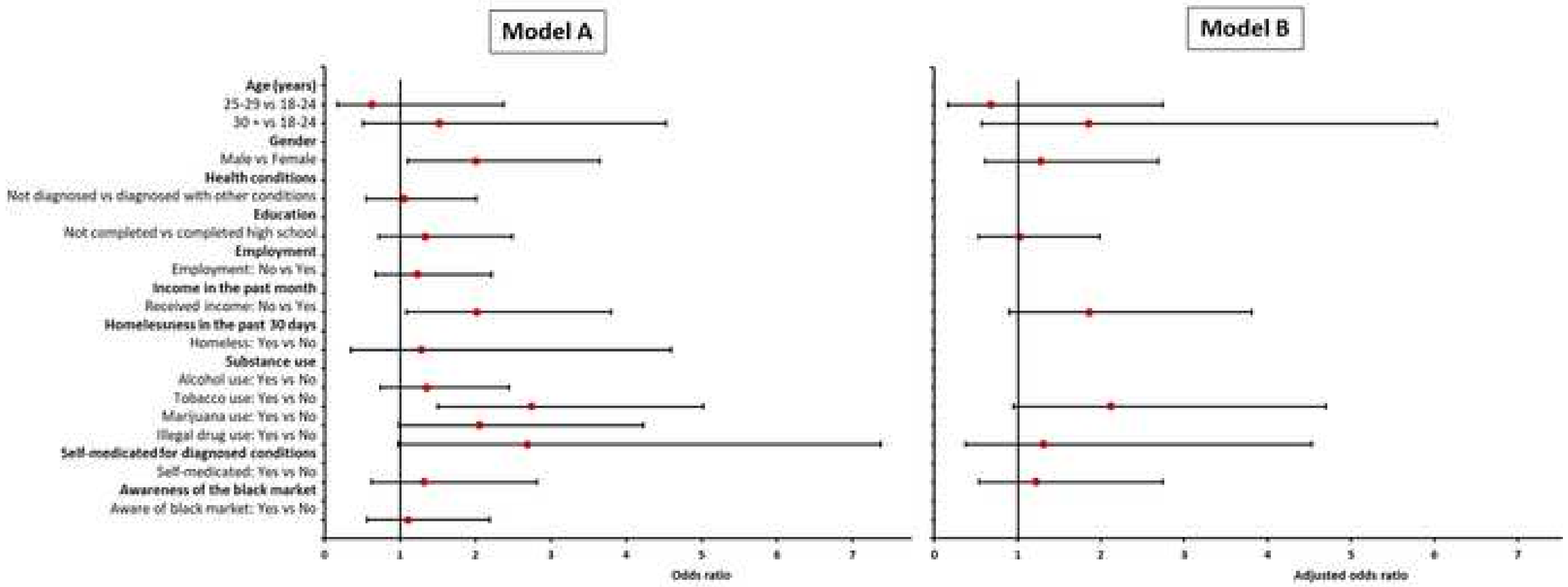

Figure 4

Click here to access/download;Figure;Fig 4.tif

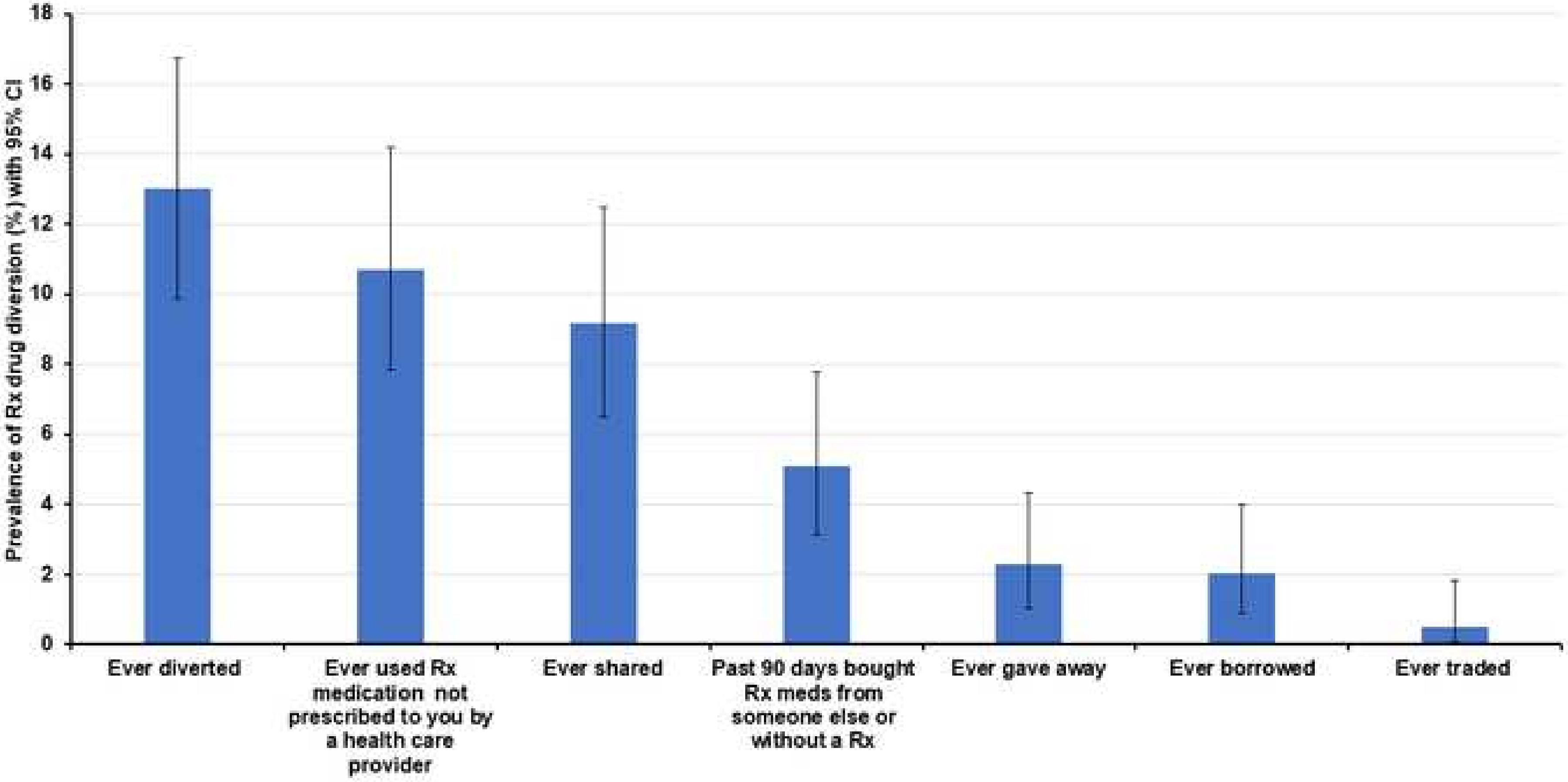

Figure 5

Click here to access/download;Figure;Fig 5.TIF

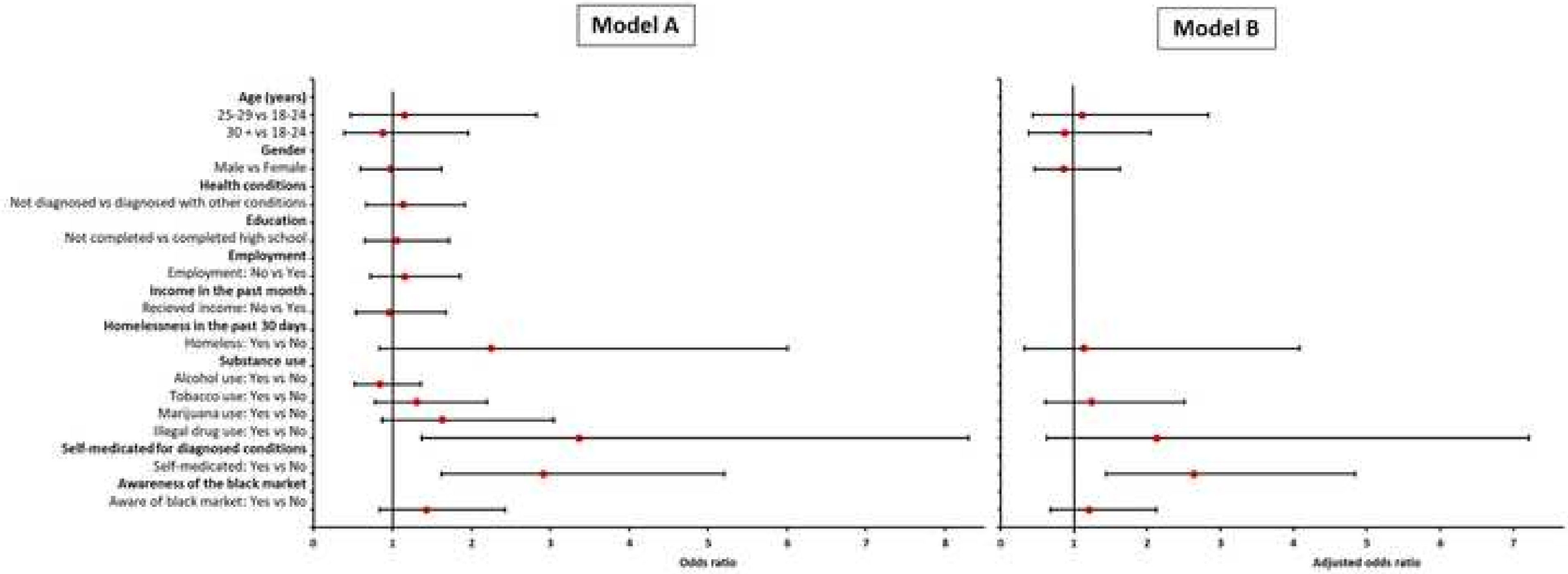

Click here to access/download
Supporting Information
Supplementary materials.docx
