# Supplementary material for: Prevalence and correlates of prescription drug diversion and misuse among people living with HIV in the eThekwini district, KwaZulu-Natal, South Africa
Source: PLoS One. 2020 Dec 16;15(12):e0243718. doi: 10.1371/journal.pone.0243718 (PMC7744047; doi:10.1371/journal.pone.0243718)
Supplement: Attachment — Submitted filename: Response to Reviewers.docx [file pone.0243718.s008.docx]

No comments from reviewer 2

| **Reviewer 4 comments** | **Response** | **Reference (from the revised manuscript with no track changes)** |
| --- | --- | --- |
| 1.  line 103: "might unfolds" should be "might unfold" | "might unfolds" changed to "might unfold" | Page 5, line 123 |
| 2.  lines 122-123: "areas with lack training" should possibly be "areas which lack tracking" | “areas with lacking training in the area” changed to “urban areas which lack tracking” | Page 6, line 145-146 |
| 3.  line 207: "would their participation affect their healthcare" should be "and participation would not affect their healthcare" | “or would their participation affect their healthcare” changed to “and participation would not affect their healthcare” | Page 10, line 236 |
| 4.  line 280: mention these are exact 95% confidence intervals | Suggestion taken | Page 13, line 316 |
| 5.  Table 1: authors should carefully check numbers and p-values as I found a number of incorrect calculations. Examples include the percentage for 18-24 years in the Total sample (which should be 10% not 7%), the percentage of completed high school and tertiary studies in the did not misuse prescription drugs group (which should be 10% not 26%), the numbers of homeless add to 18, not 17 when looking at the yes/no columns for both divert and misuse prescriptions, the p-value for ever used alcohol comparing those that diverted and those that did not seems wrong (based on the numbers provided), I get a much smaller p-value for ever used tobacco in those that diverted vs not, the numbers of ever used marijuana in the diverted and did not divert columns add to 56 not 55. | Analysis were redone to correct errors in Table 1  Age 18-24 year is 7%  the percentage of completed high school and tertiary studies in the did not misuse prescription drugs group is 10%  homeless participants were 18  p-value for ever used alcohol comparing those that diverted and those that did not = 0.410  p-value for ever used tobacco in those that diverted vs not = 0.001  the numbers of ever used marijuana in the diverted and did not divert columns add to 55 | Page 16-22 |
| 6.  It is recommended that in the text (lines 339, 360, 390) and S2 and S3 tables, the authors remind the readers what model A and model B mean. At a minimum, there should be a note for S2 and S3 tables that defines these models. | Suggestion taken | Page 14, line 321-328 |
| 7.  lines 350-356 are all results that have extremely high p-values, so reporting a 20% or 50% increase in odds is misleading. There is no evidence in the data to suggest that there is any increased chance of diverting PM based on these characteristics. I am fine with keeping the marijuana and illegal drugs findings, since with the 95% confidence interval, the readers can see that the lower limit for the CI is just under 1. | all results that have extremely high p-values, so reporting a 20% or 50% increase in odds were removed except for marijuana and illegal drugs | Page 23, line 385-393 |
| 8.  lines 365 and 367, remove "among the participants who misused drugs", because the authors are reporting percentages out of the entire sample not just those who misused drugs. | Changed to “among all the participants in the study”,… | Page 24, line 403 |
| 9.  lines 321-385: it is misleading to say that these have higher odds of misusing PM, since the results all have p-values >0.1 | Misleading statement was deleted | Page 24-25, line 417-421 |

| **Additional Editor Comments** | **Reference (the document with track changes)** |
| --- | --- |
| I have concerns regarding the definitions of prescription drug abuse, self-medication, and diversion. | The definitions were updated according to the National Institute on Drug Abuse  Page 3-4, line 66-89 |
| What drugs specifically are you inquiring about? e potential for misuse or abuse and the implications for HIV prevention and treatment would apply only to specific classes of prescription drugs. Were these assessed? | Prescription drugs inquired about in the study were sedatives, stimulants, analgesics, antibiotics, antiretroviral drugs and any emerging prescription drugs with potential of being diverted, misused or abused.  For example:  Any sedatives (tranquilizers) such as Xanax, Valium, Ativan, Klonopin, Soma, Barbiturates or others   - For euphoric effects such as excitement and happiness   Any stimulants such as Adderall, Ritalin, Adipex or any other stimulants   - For increasing the ability to concentrate, boost feelings and performance enhancing   Any analgesics (painkillers) such as Opioids, Codeine, Tramadol, percocet, Vicodin, OxyContin, , Demerol, Darvon, Demerol, Morphine, Methadone, Fentanyl, Pentazocine   - For reducing pain or feeling high   Any antibiotics such as amoxicillin, ampicillin, cloxacillin, metronidazole, co- trimoxazole, ciprofloxacin, or others  Any ARVS  **Were the prescription drugs assessed?**  There was a limitation in the study since most participants had low knowledge and awareness of different types of prescription drugs. Participants were unable to specify the exact name of the prescription medication. |
| the definition of abuse is not in line with the substance use literature and should be reconsidered. | Definition was updated according to the National Institute on Drug Abuse  Page 4, line 79-86 |
| Please clarify that 100% response rate means that all persons approached to participate agreed and completed the survey. | This was updated. Page 15, line 359-360 |
| The need for careful editing, as some numbers appear incorrect and there are several typographical and grammatical errors throughout the manuscript that remain in this revision. In this revision I would recommend adhering to commonly accepted terms when referring to people who use drugs | Grammarly was used to improve the manuscript.  In the past, people who used drugs were called “addicts.” Current appropriate terms are people who use drugs and drug users. <https://www.drugabuse.gov/publications/media-guide/glossary> |
