# Supplementary material for: Prevalence and correlates of prescription drug diversion and misuse among people living with HIV in the eThekwini district, KwaZulu-Natal, South Africa
Source: PLoS One. 2020 Dec 16;15(12):e0243718. doi: 10.1371/journal.pone.0243718 (PMC7744047; doi:10.1371/journal.pone.0243718)
Supplement: Attachment — Submitted filename: Response to Reviewers.docx [file pone.0243718.s009.docx]

| **Academic Editor comments** | **Response** | **Reference (from the revised manuscript with no track changes)** |
| --- | --- | --- |
| 1) The legends (or footnotes) for Figures 2 and 4 should specify the difference between Model A and Model B | Description of model A and B were explained in the legends for bot drug diversion and misuse | Page 25, line 382-387  Page 26, line 415-421 |
| 2) In the analysis section, the statements about the selection of factors included in the model should be appropriately referenced. For example, where in lines 329-30 commenting on "known confounders" of the exposure-outcome relationship. Also, with respect to the language in this paragraph; these are associations, not effects, between the variables and the outcomes. These factors are also more appropriately termed correlates, not predictors. | Reference 40 and 41 were added  The word predictors was replaced with correlates in the manuscript title  The word effect was replaced with associations | Page 14, line 318  Page 1, line 1  Page 13, line 308-317 |
| 4) The assessment of "drug abuse" is not an accepted method of measurement, and the terminology is outdated. The idea that one could assess this type of use by directly asking about use to intentionally harm oneself lacks the understanding that addiction is a behavioral pattern in which one uses substances despite the negative consequences. I highly recommend removing this from the paper and focusing on diversion and misuse and the types of misuse examined. | Suggestion taken into consideration. All information related to drug abuse was removed from the paper. |  |
