# Supplementary material for: Prevalence and correlates of prescription drug diversion and misuse among people living with HIV in the eThekwini district, KwaZulu-Natal, South Africa
Source: PLoS One. 2020 Dec 16;15(12):e0243718. doi: 10.1371/journal.pone.0243718 (PMC7744047; doi:10.1371/journal.pone.0243718)
Supplement: Attachment — Submitted filename: Response to reviewer comments 3 April 2020.docx [file pone.0243718.s007.docx]

| **Reviewer 1 comments** | **Response** | **Reference (the document with track changes)** |
| --- | --- | --- |
| Data presented in the results section does not match the narrative (comments attached). | The narrative was revised to match the data. | Page 19, line 383-399; page 20, line 406-414; page 21, line 445-450 |
| It is also not clear if the authors are discussing diversion and misuse of ARVs, or opioids used for pain management.  Page 2, line 23-33.  It would be helpful to describe the type of drugs from the very beginning | The type of prescription drugs diverted, misused and abused were excluded in the analysis since we only had data for type of prescription drugs diverted and abused. This posed as a limitation to our study analysis to list and compare the types of prescription drugs diverted, misused or abused | Page 27, line 600-602 |
| unclear sentence | The entire sub-section of measures was revised | Page 10-12, line 244-299 |
| What does this mean? Some people participated in more than one survey? | The response rate was 100% | Page 14, line 358 |
| any drugs? ARVs? opioid-based pain medications? | The type of prescription drugs diverted, misused and abused were excluded in the analysis since we only had data for type of prescription drugs diverted and abused. This posed as a limitation to our study analysis to list and compare the types of prescription drugs diverted, misused or abused | Page 27, line 600-602 |
| still considered significant? p>0.05, and 95%CI crosses 1. Looking at the data, I would conclude that only tobacco use has increased the odds of PV diversion | The paragraph was revised.  Marijuana and illegal use were deleted since they were not significant | Page 19, line 383-393 |
| data indicates that none of the factors increased the odds | The following factors: no income, ever used tobacco, ever used illegal drugs and self-medicated had more than 20% chance of occurring  Not changed |  |
| again, it would be helpful to know which drugs. below you talk about pain management, so did they misuse pain medications? | The type of prescription drugs diverted, misused and abused were excluded in the analysis since we only had data for type of prescription drugs diverted and abused. This posed as a limitation to our study analysis to list and compare the types of prescription drugs diverted, misused or abused | Page 27, line 600-602 |
| no data shown to support the statement about increased odds | The author had taken noted that the p-values were >0.05 (no association). However, our participants with the following characteristics (homelessness, used tobacco, used marijuana and are of the black had higher chances of misusing prescription medication. |  |
| mental? | Corrected | Page 26, line 580 |

| **Reviewer 2 comments** | **Response** | **Reference (the document with track changes)** |
| --- | --- | --- |
| *I dont believe that most of the research on this topic is mostly conducted in the USA only | Studies conducted in South Africa were included | Page 5-6, line 120-142 |
| *Reference provided about studies done in other countries (China, Jordan) show findings of prescription drug misuse in the general population and not im PWLH; therefore not similar to this study's findings | Sentence was revised to indicate that similar patterns of prescription drug misuse were observed though in different population | Page 23, line 500-510 |
| *There are several studies conducted in SA that looked at use and abuse of prescription medications; but these studies are not mentioned; and the statement mentioned about the study on misuse of codeine containing medicines use is not correct | South African studies were added, and the incorrect statement was rectified | Page 23, 493-506 |
| *Description of 'misuse and abuse' is not clearly stated and that is confusing | Definition section was added | Page 3-4, line 60-81 |
| *I appreciate the acknowledgement of excluding mental health variable as a limitation; i think the authors should have looked at it as is almost impossible to talk about HIV and exclude mental health (depression, anxiety, etc) | The mental health variable was not included in the analysis since there were a lot of missing data. |  |
| *Looks like figure 2 and figure 3 is the same graph and the y-axis heading is the same, confusing | Corrected, now represented as Fig 1 and Fig 3 | Attached Fig 1 and Fig 3 |
| *There are spelling mistakes, so the authors need to re-read the manuscript to avoid this | Rigorous editing was done to the entire paper | Entire paper |
| *I would have liked to see the names of commonly misused/diverted/abused medications; to see if these medications are mostly associated with mental health illnesses (to self-medicate) | The type of prescription drugs diverted, misused and abused were excluded in the analysis since we only had data for type of prescription drugs diverted and abused. This posed as a limitation to our study analysis to list and compare the types of prescription drugs diverted, misused or abused | Page 27, line 600-602 |
| Data analysis was conducted rigourously and that is appreciated. So i think the authors should be given another chance to look at corrections and resubmit the manuscript | Thank you |  |

| **Reviewer 3 comments** | **Response** | **Reference (the document with track changes)** |
| --- | --- | --- |
| The reviewer feels that it would be extremely valuable for the authors to engage further with literature around prescription and over the counter medication in low and middle income countries, but in South Africa particularly to get a sense of what some of the concerns and issues are in this context. Examples are publications on codeine data (authors to look for are Parry, C., Dada, S. and Carney, T.) and the SACENDU data which is widely available. There are also definitely at least a few studies on opioid use and HIV, which is increasingly a problem in KZN. | South African studies were included | Page 5-6, line 120-142 |
| Definitions of misuse and abuse and diversion are also not clearly provided. Please look at the latest terminology used in substance use literature. | Definition section was added | Page 3-4, line 60-81 |
| It would have been very interesting to look at the relationship between HIV outcomes and problem prescription use among PLWH-could these types of analyses be run? | The study was self-report and most participants did not know their latest viral load. |  |
| There are some studies conducted in South Africa (one qualitative one actually conducted in KZN) that mention people may be misusing ART as well. Was this included in the current study?' | South Africa studies were added. | Page 5-6, line 120-142 |
| The results section could be presented in a more succinct way and it is difficult to interpret Figure 3 and 4. | The correct figure for Figure 4 was placed  The results section was revised as advised by other reviewers  Figure 3 (Forest plot) was not changed. Authors viewed forest plot as appropriate plots of odds ratios.  S2 Table, is a table for all the values for odds ratios |  |
| A suggestion would be to present the univariate and multiple regression models in one table-perhaps look at other literature for examples. | Regression models A and B were presented in one table for prescription drug diversion  Regression models A and B were presented in one table for prescription drug misuse | S2 table  S3 table |
| A study limitation is that this is a small sample size to be representative of eThekwini PLWH and attending healthcare services. | This was added as a limitation | Page 27, line 600-613 |
| Are there any additional comments to make around this, such as do the authors think that this is a representative sample? | The minimum sample size of 385 was determined using the formula developed by the Daniel (1982) for a finite population with an assumption of 50% prevalence. Due to limited funding for data collection the study was able to enrol 392 PLWH. | Page 27, line 600-602 |

| **Reviewer 4 comments** | **Response** | **Reference (the document with track changes)** |
| --- | --- | --- |
| 1. Figure 2: it is unclear why the confidence intervals presented in Figure 2 are not symmetric, since typical confidence intervals for proportions are symmetric. Authors should clarify how confidence intervals were computed and verify that what is presented in Figure 2 is correct. | The error bars were doubled checked and below is an example of how they were obtained.  Exact binomial tests were computed in R Studio to get obtain the 95% confidence interval. For example:  Exact binomial test  data: 89 (diverters) and 392 (sample size)  number of successes = 89, number of trials = 392, p-value < 2.2e-16  alternative hypothesis: true probability of success is not equal to 0.5  95 percent confidence interval:  0.1864918 (therefore, 23% - 18.6% = 4.35% = the lower level errors,)  0.2717666 (therefore, 27.2% -23% = 4.18% = the upper level error |  |
| 2. lines 212-216 (also in the Abstract lines 36-40) and lines 248-250: The authors are reporting percentages out of all 392 participants, which isn't necessarily wrong, but the start of the sentences ("Majority" or "More than one-third") suggest that the reader should be focused on just those with drug diversion or with drug misuse. The authors are encouraged to think about what point they really want to make and whether it is most appropriate to report percentages out of the entire sample or out of just those that had the action of interest. | Data was only presented as percentage (n). | Page 19, line 376  Page 20, line 420 and line 423 |
| 3. The authors have a tendency to overstate the findings, even when qualifying a statement by saying something wasn't statistically significant. In most of the situations, findings are not even close to being significant. Examples include lines 233-239, 241-244, 263-266, 272-275). Authors already include the ORs and 95% confidence intervals for the terms in the model in supplemental table 3, so readers can see these results.  Some of these end up being discussed in the discussion (age and aware of the black market) and highlighted in the Abstract (homeless and awareness of the black market) as findings, which is inappropriate. In the discussion, they further highlight differences between males and females, but this finding does not hold up in the adjusted model. | The results section was revised  This were removed from the abstract and discussion section  Discussion related to gender was removed | Page 2, line 48-50  Page 24-25, line 535-547 |
| 4. In the adjusted models for drug diversion (model B), the authors include more variables than are generally recommended given the number of people who had the action of interest. Standard rules of thumb for logistic regression would suggest that no more than 5 predictors should be included in a model, yet the authors include 7. | Mode B for prescription drug diversion and misuse with only five variables was redone. I  However, we found that there was no significant difference between the original model and revised model looking at the Odds ratios | **Model B, Prescription drug diversion:** **Variables** \| **Original Odds** \| **Revised Odds**; **Age (years)** \| **-** \| **-**; 25-29 vs 18-24 \| 0.68 \| 0.67; 30 + vs 18-24 \| 1.85 \| 1.87; **Gender** \| **-** \| **-**; Male vs Female \| 1.28 \| 1.29; **Health conditions** \| **-** \| **-**; Not diagnosed vs diagnosed with other conditions \| **-** \| **-**; **Education** \| **-** \| **-**; Not completed vs completed high school \| 1.03 \| **-**; **Employment** \| **-** \| **-**; Employment: No vs Yes \| **-** \| **-**; **Income in the past month** \| **-** \| **-**; Received income: No vs Yes \| 1.86 \| 1.82; **Homelessness in the past 30 days** \| **-** \| **-**; Homeless: Yes vs No \| - \| -; **Substance use** \| **-** \| **-**; Alcohol use: Yes vs No \| **-** \| **-**; Tobacco use: Yes vs No \| 2.12 \| 2.09; Marijuana use: Yes vs No \| **-** \| **-**; Illegal drug use: Yes vs No \| 1.31 \| 1.38; **Self-medicated for diagnosed conditions** \| **-** \| **-**; Self-medicated: Yes vs No \| 1.22 \| -; **Awareness of the black market** \| **-** \| **-**; Aware of black market: Yes vs No \| **-** \| **-** **Model B, Prescription drug misuse:** **Variables** \| **Original Odds** \| **Revised Odds**; **Age (years)** \| **-** \| **-**; 25-29 vs 18-24 \| 1.11 \| 1.09; 30 + vs 18-24 \| 0.88 \| 0.88; **Gender** \| **-** \| **-**; Male vs Female \| 0.86 \| 0.86; **Health conditions** \| **-** \| **-**; Not diagnosed vs diagnosed with other conditions \| **-** \| **-**; **Education** \| **-** \| **-**; Not completed vs completed high school \| **-** \| **-**; **Employment** \| **-** \| **-**; Employment: No vs Yes \| **-** \| **-**; **Income in the past month** \| **-** \| **-**; Received income: No vs Yes \| **-** \| **-**; **Homelessness in the past 30 days** \| **-** \| **-**; Homeless: Yes vs No \| 1.14 \| 1.08; **Substance use** \| **-** \| **-**; Alcohol use: Yes vs No \| **-** \| **-**; Tobacco use: Yes vs No \| 1.24 \| 1.23; Marijuana use: Yes vs No \| **-** \| **-**; Illegal drug use: Yes vs No \| 2.13 \| 2.32; **Self-medicated for diagnosed conditions** \| **-** \| **-**; Self-medicated: Yes vs No \| 2.64 \| 2.70; **Awareness of the black market** \| **-** \| **-**; Aware of black market: Yes vs No \| 1.21 \| **-** |

| **Additional information not suggested by reviewers but the author thought they were important** | **Reference (the document with track changes)** |
| --- | --- |
| Study setting | Page 7, line 165-176 |
| Information related to the NGO | Page 8, line 200-205 |
| Ethical consideration | Page 9, line 233-243 |
